# Genome-wide epigenetic dynamics during postnatal skeletal muscle growth in Hu sheep

**DOI:** 10.1038/s42003-023-05439-0

**Published:** 2023-10-23

**Authors:** Yutao Cao, Yue Ai, Xiaosheng Zhang, Jinlong Zhang, Xianlei Long, Yaning Zhu, Linli Wang, Qingyi Gu, Hongbing Han

**Affiliations:** 1 Beijing Key Laboratory of Animal Genetic Improvement, College of Animal Science and Technology, China Agricultural University, Beijing, China; 2 National Engineering Laboratory for Animal Breeding, College of Animal Science and Technology, China Agricultural University, Beijing, China; 3 Key Laboratory of Animal Genetics, Breeding and Reproduction of the Ministry of Agriculture and Rural Affairs, College of Animal Science and Technology, China Agricultural University, Beijing, China; 4 Tianjin Key Laboratory of Animal Molecular Breeding and Biotechnology, Tianjin, China; 5 Institute of Automation, Chinese Academy of Sciences, Beijing, China; 6 Frontiers Science Center for Molecular Design Breeding (MOE), China Agricultural University, Beijing, China

**Keywords:** Epigenomics, DNA methylation

## Abstract

Hypertrophy and fiber transformation are two prominent features of postnatal skeletal muscle development. However, the role of epigenetic modifications is less understood. ATAC-seq, whole genome bisulfite sequencing, and RNA-seq were applied to investigate the epigenetic dynamics of muscle in Hu sheep at 3 days, 3 months, 6 months, and 12 months after birth. All 6865 differentially expressed genes were assigned into three distinct tendencies, highlighting the balanced protein synthesis, accumulated immune activities, and restrained cell division in postnatal development. We identified 3742 differentially accessible regions and 11799 differentially methylated regions that were associated with muscle-development-related pathways in certain stages, like D3-M6. Transcription factor network analysis, based on genomic loci with high chromatin accessibility and low methylation, showed that *ARID5B*, *MYOG*, and *ENO1* were associated with muscle hypertrophy, while *NR1D1*, *FADS1*, *ZFP36L2*, and *SLC25A1* were associated with muscle fiber transformation. Taken together, these results suggest that DNA methylation and chromatin accessibility contributed toward regulating the growth and fiber transformation of postnatal skeletal muscle in Hu sheep.

## Abstract (web summary)

Multi-omic profiling of postnatal muscle development in Hu sheep from 3 days to 12 months of age highlights the epigenetic factors involved in regulating skeletal muscle growth.

## Introduction

The postnatal development of skeletal muscle is characterized by an increase in cross-sectional area (CSA) and a decrease in the proportion of slow-twitch muscle fibers (also known as type I muscle fibers). Although muscle fibers can be hypertrophic without satellite cell fusion and muscle nucleus accretion^1^, sustained hypertrophy without the addition of muscle satellite cells will be attenuated, accompanied by excessive extracellular matrix accumulation^2^. Muscle satellite cells undergo proliferation to further fuse with myoblasts to form multinucleated muscle fibers. This process largely determines the efficiency of protein synthesis and the potential of hypertrophy^3^. Furthermore, it has been reported that functional 3D muscle tissue made from human primary myoblasts shows the characteristics of myotube hypertrophy and functional maturity within 4 weeks of culture in vitro^4,5^.

Fast-twitch muscle fibers (also called type II muscle fibers) rely on anaerobic respiration (glycolysis alone). Slow-twitch fibers prefer oxidative metabolism and are fatigue-resistant^6^. It has been proved that transcription factors (TF) *NFATC1*^7^, *MyoD*, and *MEF2C*^8,9^ regulated muscle fiber type transformation and metabolic homeostasis. PGC-1α cooperates with *MEF2* to activate gene transcription and act as the target of calcineurin signal transduction. Calcineurin signal transduction controls the expression of genes related to slow-twitch fiber formation. Similarly, *FoxO1* stimulates fast-twitch fiber formation and impairs oxidative metabolism of muscle, at least in part by inhibiting the calcineurin pathway^10^.

Epigenetics can affect postnatal muscle development by regulating transcription and translation efficiency without changing the DNA sequence. For example, miR-208b specifically targets the E-protein family member transcription factor 12 (*TCF12*) to mediate the proliferation and differentiation of myogenic cells and targets *FNIP1* to stimulate fast and slow-twitch fiber conversion^11^. Acetylation of *FoxO1* and *FoxO3* leads to muscle atrophy, while deacetylation promotes muscle fiber regeneration^12^. Comparative epigenetics were also used to investigate meat-quality-related methylation patterns^13–15^. Despite the lack of sufficient evidence on hypertrophy and muscle fiber type transformation, the spatiotemporal regulation of gene transcription may be regulated by epigenetics^16,17^.

Studies from the model animal have focused on muscle regeneration^18^, fetal muscle fiber proliferation^19–21^, and compartmentalized regulation of muscle fibers^22^. Although a few studies have focused on postnatal epigenetic development of muscle^23,24^, to our knowledge, there are no reports systematically integrating DNA methylation, chromatin accessibility, transcriptome, and related phenotype data to study postnatal muscle hypertrophy and fiber transformation. With the valuable phenotype data and publicly available datasets, our study aims to explore the postnatal genome-wide landscapes of DNA methylation and chromatin accessibility of skeletal muscle for Hu sheep and to analyze their interactions as well as the temporal regulation of the transcription factor binding sites that are exposed via epigenetic modification.

## Results

### Global changes of the transcriptome during postnatal muscle growth

**Fig. 1:**
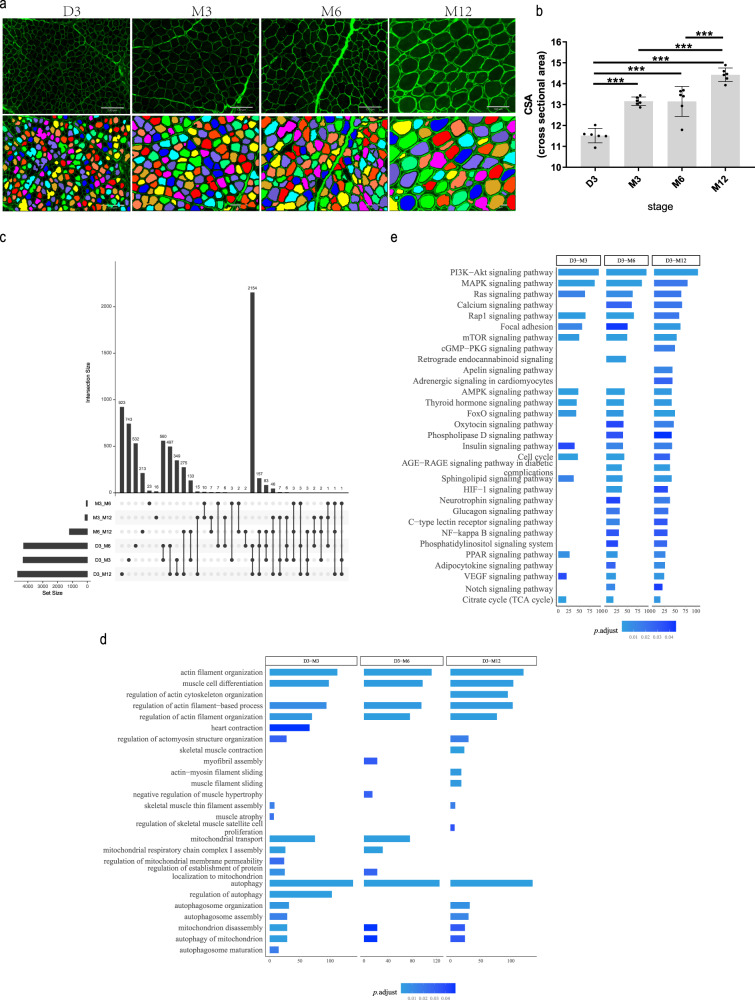
Global changes of the transcriptome during postnatal muscle growth. **a** Skeletal muscle tissues from the six Hu sheep were processed for histological and morphometric analysis. Representative photomicrographs of WGA-stained muscle sections and their digitally processed image from D3 to M12. Scale bar: 130 µm (*n* = 6). **b** Quantification of average muscle fiber CSA of the seven Hu sheep from D3 to M12. ***P < 0.0001; *n* = 6. **c** Upset plot summarizing the distribution of DEGs in all comparison groups (*n* = 6). **d** The GO items related to muscle growth of comparison groups referring to D3 (D3-X) in (**c**). **e** KEGG pathways of D3-X groups DEGs in (**c**). Error bars represent mean values ± SD. CSA cross-sectional area, WGA Wheat Germ Agglutinin, DEGs differentially expressed genes, SD standard deviation.

The number of myofibers is thought to remain fixed following birth, muscle fiber hypertrophy growth is the primaryway of muscle growth. The average CSA is generally used to measure the hypertrophy of skeletal muscle fibers. To systematically identify the growth of postnatal skeletal muscle, the average CSA of muscle fiber was measured from the quadriceps femoris of six Hu sheep at 3 days (D3), 3 months (M3), 6 months (M6) and 12 months (M12) after birth. Compared with D3, the average CSA of M3, M6 and M12 increased significantly, *P* value < 0.001, indicating that the hypertrophic growth of skeletal muscle fibers is evident in postnatal growth. On the contrary, there was no significant difference between the average CSA between M3 and M6, *P* value > 0.05, although the CSA of some muscle fibers increased, the CSA of some muscle fibers did not increase significantly, indicating that the hypertrophic growth of skeletal muscle fibers was not significant at this stage, (Fig. 1a, b). From M6 to M12, the average CSA of fibers was increased further (Fig. 1a, b). Muscle fibers are the main components of skeletal muscle tissue, followed by some other components such asconnective tissue. These data suggested two growth peaks of skeletal muscle of Hu sheep occurred  at D3-M3 and M6-M12. The growth of connective tissue might predominate, and the hypertrophy of muscle fibers is relatively weak at M3-M6.

Next, 6865 differentially expressed genes (DEGs) were obtained based on all six combinations of four stages (Supplementary Fig. 1a and Supplementary Data 2). In other words, all possible comparisons were enumerated to include genes as much as possible. D3-M3, D3-M6, and D3-M12 shared 31.3% (2154/6865) of total DEGs, while overlapped DEGs of any two of the three groups shared more than 44.2%. The overlapping rate between D3-M3 and D3-M6 was the highest (55.4%, 3066/5535, Fig. 1c), while, interestingly, M3-M6 only had 77 DEGs. This may be due to genes becoming more variable as muscles grow ( Statistical *P* value is the only hard threshold for a DEG). When D3 acted as a baseline (D3-M3, D3-M6, and D3-M12), the DEGs number and muscle-related pathways outnumbered the rest comparisons. Many pathways overlapped on the most prominent changes as muscle matured (Fig. 1d, e), such as the citrate cycle (Fig. 1e), actin filament organization, muscle cell differentiation, autophagy, mitochondrion disassembly, etc. In addition, some signs of functional maturity were found exclusively in D3-M6 and D3-M12. Myofibril assembly and thin filament assembly preferred to be in D3-M3 and D3-M6 while pathways like skeletal muscle contraction and actin-myosin filament sliding were significant only at D3-M12 (Fig. 1d). As for KEGG pathways, D3-M6 and D3-M12 included signal pathways such as calcium signal pathway, oxytocin signal pathway, and HIF-1 signal pathway, while the adrenaline signal pathway of cardiomyocytes was only observed in D3-M12 group (Fig. 1e).

**Fig. 2:**
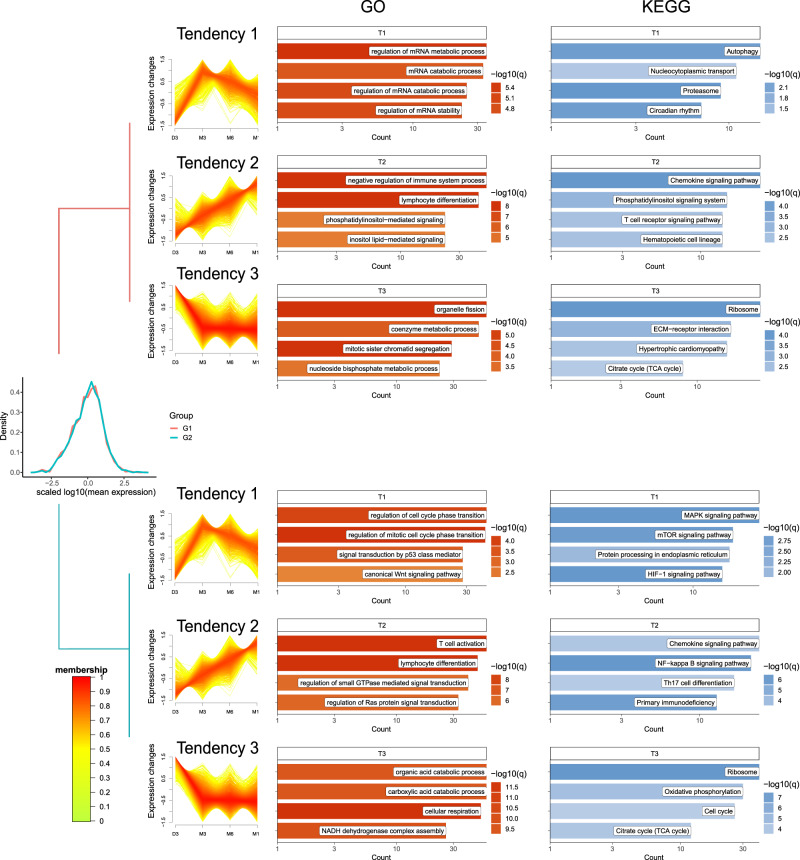
Main biological processes involved in postnatal muscle growth. Expression distribution of G1 and G2 group and first 4 significant items of each tendency (T1–T3) within the two groups. The *x* axis represented scaled expression levels and the *y* axis represented density in the line plot, with the orange color mapping membership scores of the member genes indicating their distance.

Although consecutive comparisons within neighbor stages (D3-M3, M3-M6, M6-M12) make sense to depict muscle growth in part, the enrichment results had no direct clues of muscle development as comparisons with D3. To better understand the expression pattern of DEGs and the biological processes featured in postnatal growth of skeletal muscle, all 6865 DEGs were clustered into two groups (G1 and G2) based on the Spearman co-efficiency between DEGs expression and CSA of muscle fibers. These two groups of genes were further assigned into 3 distinct tendencies (T1–T3, Fig. 2 and Supplementary Fig. 1b), respectively, in terms of the biological processes they were involved. Although there was no obvious difference between the distributions of gene expression (Fig. 2) from G1 and G2 as well as that of correlation with CSA (Supplementary Fig. 1c) to our surprise, tendencies of G1 and G2 converged into three similar biological pathways, with four representative terms selected from both GO and KEGG results for each trend (Fig. 2).

After summiting at M3 in G1 and G2, the first tendency T1 decreased gradually ever since, which indicates the potential activities of protein synthesis. Most pathways involved mRNA and protein metabolism, like KEGG items in both groups, and cell proliferation, like regulation of cell cycle phase transition in GO terms of G2; The second tendency T2 kept soaring since D3, which was involved in lymphocyte differentiation and signal cascade in G1 and G2; the third tendency T3, a mirror image of T1, exhibited a complementary relationship between cell proliferation and cell respiration: the T3 in G1 was contributed to energy-consuming processes such as organelle division, mitotic sister chromatid segregation, and nucleoside bisphosphate metabolic process, while its counterpart in G2 was mainly involved in the organic acid catabolic process, cell respiration, and NADH dehydrogenase complex assembly, etc.

### Chromatin accessibility dynamics during the growth of postnatal muscle

**Fig. 3:**
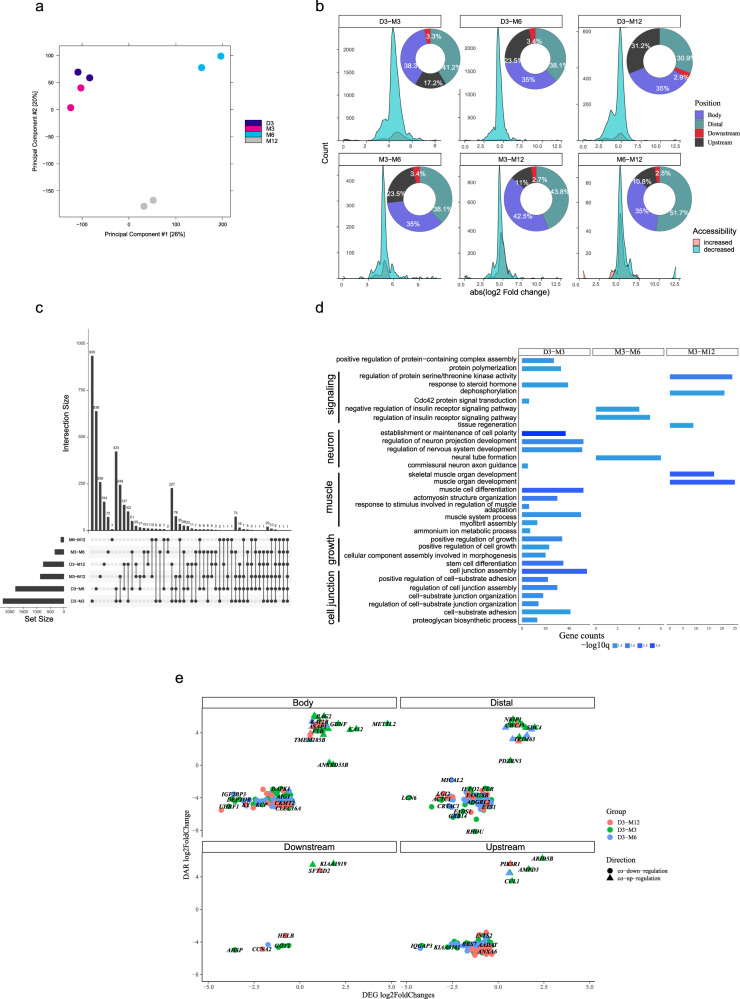
Chromatin accessibility dynamics during the growth of postnatal muscle. **a** PCA plot summarizes the relationship among ATAC-seq samples. **b** The density plot summarizes the signal difference (of mean read counts) of DAR and a pie plot for their distribution on different genomic features (gene body, distal regions, downstream and upstream of the gene) in all comparison groups. The genome was divided into four regions: upstream region (including TSS-3kb and 5’ UTR), gene body, downstream region (downstream and 3’ UTR), and distal region (distal intergenic region). Taking D3-M3 as an example, the increased red area curve indicates stronger accessibility of the DARs in M3 (logM3-logD3 > 0) and, 41.2% DAR lies in distal intergenic regions. **c** Upset plot summarizing the distribution of DAG in all comparison groups. **d** GO items for all comparison groups in (**c**). The same category was grouped and labeled aside. **e** Genes regulated by chromatin accessibility (or transcriptionally consistent DAR) were divided into two groups: co-upregulated genes (in the first quadrant) and co-downregulated genes (in the third quadrant) at different genomic feature facets. Co-regulated genes at specific stages (D3-M3, D3-M6, and D3-M12) were in different colors. DAR differentially accessible regions, DAG differential accessible genes, TSS transcription start site, UTR untranslated region.

To capture chromatin accessibility changes during postnatal muscle development, the assay for targeting accessible chromatin with high-throughput sequencing (ATAC-seq) was used. The average number of accessible peaks at four developmental stages (D3-M12) were, respectively, 30779 ± 11995, 22647 ± 1416, 18553 ± 8942 and 24185 ± 5981. To obtain high-quality data, the irreproducibility discovery rate method (IDR) was used to screen high-confidence open chromatin peaks in three replicate samples, with a total of 461 annotated genes (Supplementary Fig. 2a and Supplementary Data 3). Interestingly, the open chromatin peaks decreased gradually from 50% to 10% with the development of postnatal muscle in CpG island (Supplementary Fig. 2b). The principal components analysis (PCA) showed the four stages (D3, M3, M6, and M12) were distinguished completely based on all 3742 differentially accessible regions (DARs) (Fig. 3a). Similar to DEGs, six comparison groups of DARs were obtained from all six possible combinations of four stages (Fig. 3b, c). As expected, D3 had the most peaks, while the difference in peak counts was the least between M6 and M12.

We calculated the log fold changes of mean read counts between comparison groups and displayed the distribution of DARs which gain or lose accessibility as muscle grew. Taking D3-M3 as an example, the increased red area curve indicates stronger accessibility of the DARs in M3, while the decreased cyan area curve indicates weaker accessibility of the DARs in M3. Decreased DARs of all six possible comparison groups accounted for 64–97% of the total differential open signal peaks (Fig. 3b). By contrast, 3–36% of the DARs increased their accessibility as muscle growth (Fig. 3b). To analyze the source of DAR, we divided the genome into four regions: upstream regions (including 3 kb upstream of the transcription start site (TSS-3kb) and 5’ untranslated regions (UTR)), gene body (gene-coding regions), downstream regions (Downstream and 3’ UTR above), and distal regions (distal intergenic regions). The distribution proportion of DARs on the genome was calculated for six comparison groups: distal regions (31–52%), gene body (35-43%), upstream regulatory regions (11–31%), and the downstream counterpart (3%) with the lowest enrichment level (Fig. 3b). Interestingly, when compared to the D3 stage, the DARs located in upstream regions of genes exhibited greater accessibility differences as muscle grew, while the relative proportion of DARs located in distal intergenic regions decreased.

Significant GO pathways of DARs, were only found in the D3-M3, M3-M6, and M3-M12 groups (Fig. 3d). Muscle-related pathways were found in D3-M3 and M3-M12 groups, especially the muscle organ development in M3-M12. Besides, the regulation of steroid and insulin hormone, neuron activities and kinase activities could be found in D3-M3, M3-M6, and M3-M12 groups, respectively. These data highlighted the important roles of epigenetic modifications in the regulation of hormone secretion, neuron activities, and muscle systems during the growth of postnatal muscle.

**Fig. 4:**
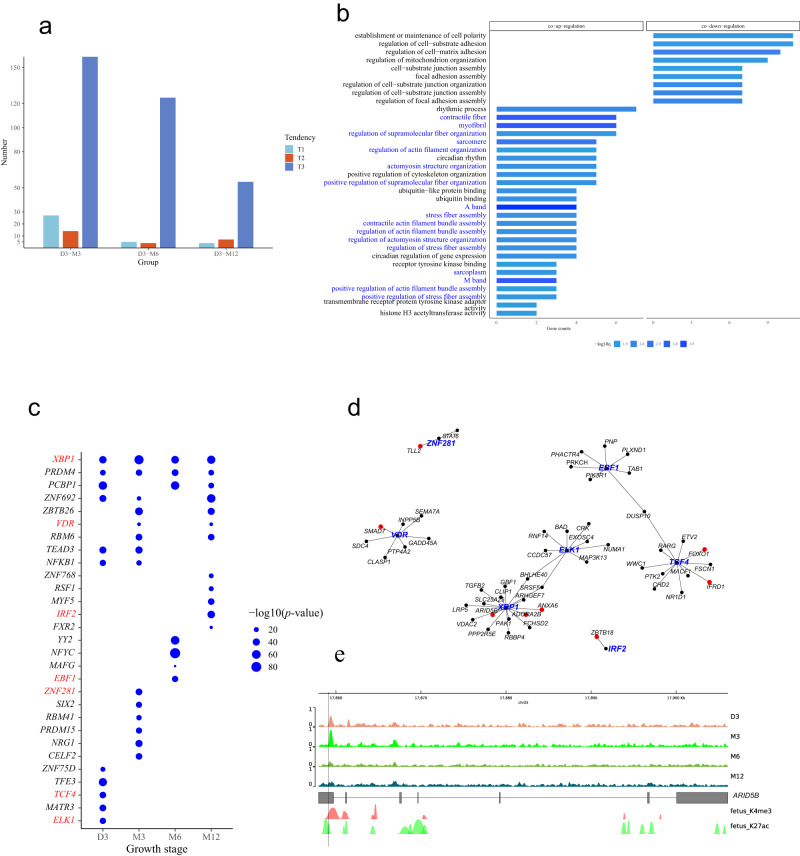
Gene network regulated muscle growth via chromosome accessibility. **a** Bar plot summarizing the distribution of genes regulated by enhancers on three expression tendencies (T1–T3). **b** GO results for co-upregulated and co-down-regulated genes. Muscle-related pathways were highlighted in blue. **c** Identification of potential TF binding to the motif of IDR peaks at different stages. Each TF was validated by the expression matrix of RNA-seq. The dot size represents the significance of TF. TF found in TF-target pairs were in the red. **d** TF-targets networks of IDR (IDR network) based on (**c**) (first-level nodes, in blue) and genes (second-level nodes, in red or black) from (**a**). Red targets represent genes in muscle-related pathways of (**a**). **e** Genome browser of *ARID5B* in IDR network, showing accessible peak signal tracks of four stages, gene structure, as well as the annotations of histone modifications. GCF_000298735.2 was used for genome annotation. Dashed lines indicated where DAR lay (*n* = 3). IDR irreproducible discovery rate, TF transcription factor, DAR differentially accessible regions.

The chromatin accessibility can directly contribute to gene transcription. The genes regulated by chromatin accessibility (or transcriptionally consistent DARs) were defined as genes that shared the same sign of fold change between DARs and DEGs in the same comparison group (Fig. 3e). For example, in the first quadrant, the co-upregulation genes in a triangle shape exhibited stronger accessibility and increased transcription levels in certain groups. Most co-upregulated genes came from D3-M3 groups. In addition, according to the expression tendencies of DEG, transcriptionally consistent DAR has a majority in the T3, whose significant pathways were related to cell proliferation and cell respiration(Fig. 4a).

Despite various genes in different facets, co-downregulated genes outnumbered their counterparts: most of the genes regulated by chromatin accessibility decreased their DAR signal intensity and downregulated gene expression; only a few genes showed DAR signal enhancement and gene expression upregulation; Next, their GO enrichment results (Fig. 4b) showed the co-downregulated genes in D3-M6 and M3-M12 groups are involved in establishment or maintenance of cell polarity. Although accounting for a smaller proportion, transcriptionally co-upregulated genes accounted for most of the significant pathways as well as muscle structure-related pathways (blue text), such as sarcomere, A band, M band, and actomyosin structure organization.

To explore the effect of TF on postnatal muscle development, a regulatory network of TF-target genes (IDR network) was constructed for chromatin accessibility-regulated genes according to the motif results (Fig. 4c) and the TF database. To reduce the false positive, the TF was predicted by the ab initio method and further validated by the RNA-seq expression data. A total of 18 pairs of regulatory relationships between 7 TF and 74 targets were obtained (Fig. 4d): *IFRD1* was found in the muscle cell differentiation pathway; *ADORA2B*, *ANXA6*, *FOXO1* were found in the muscle system process; *TLL2*, *ARID5B*, and *SMAD7* were found in muscle organ development pathways (Fig. 4b). Among these highlighted genes, the transcription level of *ARID5B* in the M6 stage received maximum upregulation and was roughly 5.7 times (log2(M6/D3) ≈ 2.5) higher than that in the D3 stage (Fig. 3e). The most significant open chromatin peak of the *ARID5B* gene is on the 5’UTR of XM_004021393.3 transcripts (Fig. 4e).

### Genome-wide DNA methylation pattern during postnatal muscle growth

**Fig. 5:**
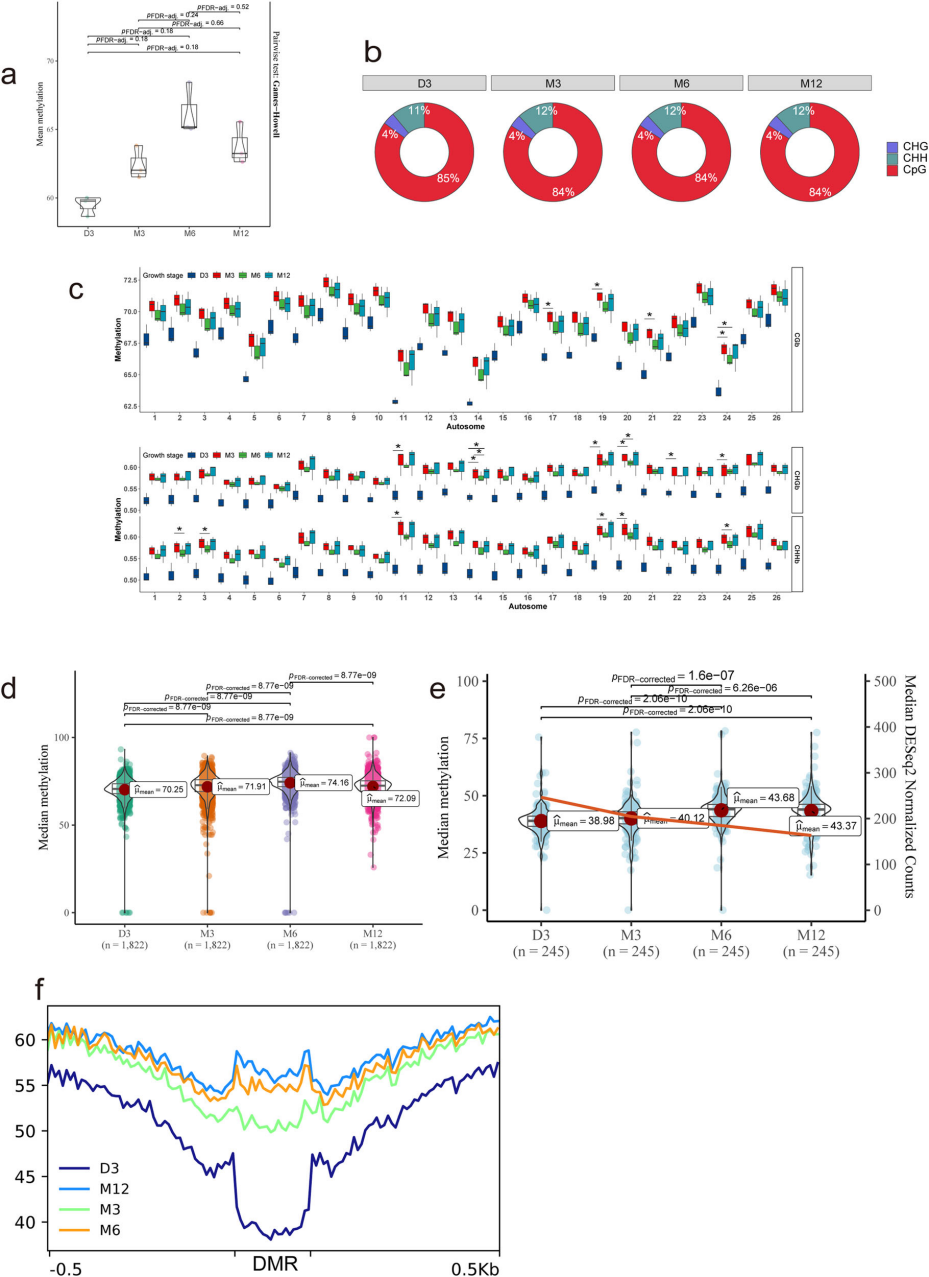
Genome-wide dynamic patterns of DNA methylation during postnatal muscle growth. **a** Box plot summarizing mean genome-wide methylation at the sample level. **b** Proportion of three types of methylated cytosine contexts across four stages: CHG, CHH, and CpG. **c** Box plot summarizing mean genome-wide methylation at the chromosome level. “*” means a significant difference (*P* < 0.05, adjusted by FDR) between the two stages. Violin plot for methylation of high methylation domains (HMD, **d**) and low methylation regions (LMR, **e**). The line in the LMR violin plot represents the median expression of genes with LMR (right *y* axis). Groups showing a significant difference were labeled with exact *P* value. **f** Line plot exhibiting mean methylation of flank sequence (DMR ± 500 bp) and DMR across four stages. Boxes or violin represent 25th to 75th percentile and whiskers represent minimum-maximum. Horizontal lines and points within boxes represent the median and mean respectively (*n* = 3). DMR differential methylation regions, HMD high methylation domains, LMR low methylation regions.

To discover the effects of DNA methylation on postnatal muscle development, whole-genome bisulfite sequencing (WGBS) was applied to evaluate DNA methylation levels of the global genome. The WGBS data achieved a 95% conversion rate, over 94% of the whole-genome coverage rate, and 89% of the alignment rate. The correlation degree among all samples could be well-distinguished by the four developmental stages (D3-M12) (Supplementary Fig. 3a). In other words, the intra-group correlation coefficient of repeated samples is higher than that of the within-group. The significant tendency in DNA methylation level could be found among D3, M6, and M12 (Fig. 5a, adjust *P* value 0.18); the average DNA methylation rates of the three contexts of methylated cytosine (mC) were 84.56 ± 0.55% (CG), 3.62 ± 0.09% (CHH) and 11.81 ± 0.46% (CHG) (Fig. 5b). However, there are significant differences in chromosome level, and most of them occur in the D3-M3 stage (Fig. 5c).

Three DNA methylation types were further assigned according to the genome-wide DNA methylation level (Supplementary Fig. 3b): high methylated domains (HMD), partially methylated domains (PMD), and low methylated regions (LMR). In summary, as muscle grew, DNA methylation levels went up except for the drops of PMD in M3 and HMD in M12. The results showed that the same DNA methylation type was changed significantly in the four developmental stages, such as HMD (Fig. 5d) and LMR (Fig. 5e). The median DNA methylation of HMD increased steadily and fell at M12. The LMR also showed a similar trend, but there was no significant drop at M12 (Fig. 5d). In addition, there was a weak negative correlation between the median DNA methylation and the expression of genes in LMR (spearman coefficient–0.1, *P* value 0.0026) (Fig. 5e).

Differential methylation regions (DMR) were defined as consecutive sections with significantly different methylation among comparison groups and were identified for all six possible combinations of four stages. The average DNA methylation of DMR (*n* = 11,799) was generally lower than that of its adjacent region (DMR ± 500 bp); Besides, the average methylation of DMR increased at a decelerated speed since D3 (Fig. 5f). Besides, the GO enrichment results of the annotation genes of DMR showed that the host genes of DMR were closely related to muscle development (Supplementary Fig. 4). In particular, the D3-X groups (D3 served as the reference, including D3-M3, D3-M6, and D3-M12) enriched pathways such as muscle system process, actin filament organization, and myofibril assembly.

**Fig. 6:**
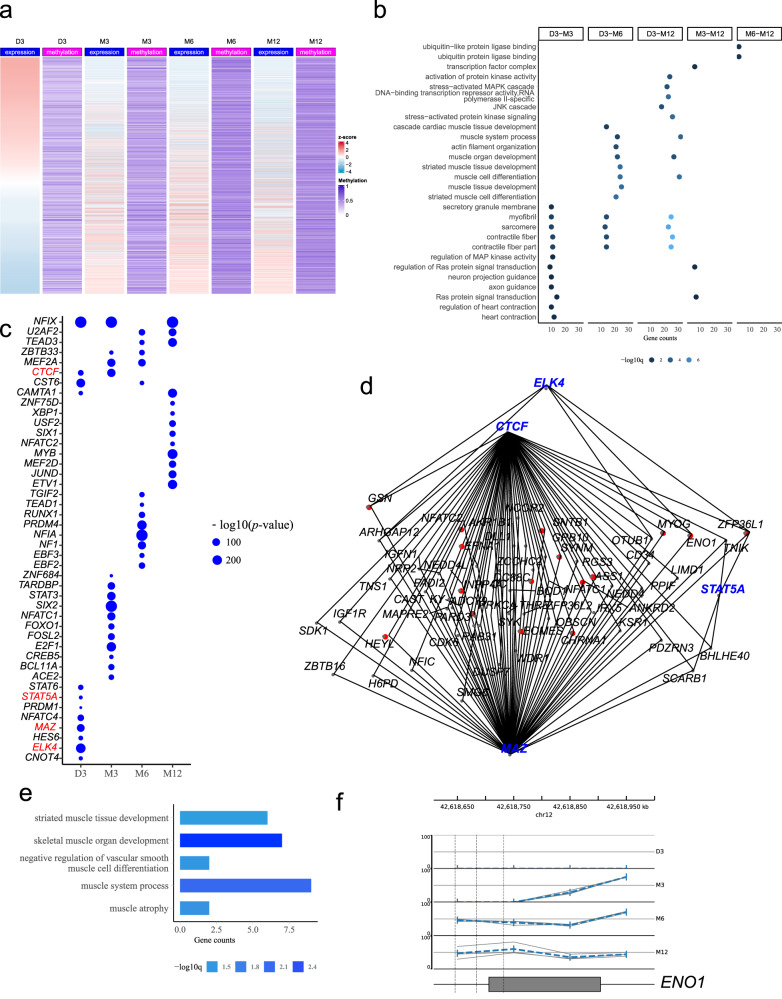
Gene network of muscle growth regulated via DNA methylation. **a** Heatmap for gene expression (red-blue bar, red indicates high expression while blue means low expression) and their methylation at DMR (purple bar, the deeper purple, the higher DNA methylation) across four stages (using the groups referring to D3). **b** GO items for intersection genes of DMR and differentially expressed genes (DEGs). **c** Identification of potential transcription factors (TF) binding on LMR. Each TF was validated by the expression matrix of RNA-seq. The dot size represents the significance of TF. TF found in TF-target pairs were in the red. **d** TF-targets networks of LMR (LMR network) based on (**c**) (first-level nodes, in blue) and genes (second-level nodes, in red or black) from (**b**). Red targets represent genes in muscle-related pathways of (**e**). **e** GO items for LMR network. **f** Genome browser of *ENO1* in LMR network, showing methylation level tracks of four stages and gene structure. GCF_000298735.2 was used for genome annotation. Dashed lines indicated where DMR lay (*n* = 3). DMR differential methylation regions, LMR low methylation regions.

To explore the relationship between DMR and their host genes’ expression, the intersection of the host genes of DMR and DEGs were defined as DMR-regulated genes and selected from D3-X comparison groups. Their DNA methylation and transcription levels were depicted as muscle development (Fig. 6a). The results showed that despite the level of DMR generally increasing as muscle grew, the expression level of the corresponding genes was not linearly negatively correlated with the average DNA methylation level of their DMR. Most genes followed higher methylation and lower transcription patterns along with the development of muscle. However, the upregulated genes are also accompanied by increased DNA methylation of DMR (Fig. 6a), which may indicate that other regulatory mechanisms are involved. Similarly, the correlation between gene expression and DNA methylation of DMR was depicted for the M3-M6, M3-M12, and M6-M12 groups (Supplementary Fig. 5a-c). GO enrichment further highlighted the roles of these genes in postnatal muscle development. In D3-M3, D3-M6, and D3-M12 groups, the enrichment biological processes were directly related to muscle development including the contractile fiber, contractile fiber part, myofibril, sarcomere and so on (Fig. 6b). The muscle system process, muscle cell differentiation, and muscle organ development appeared simultaneously in the D3-M6 and D3-M12 groups, respectively (Fig. 6b). In addition, the ubiquitin ligase binding and ubiquitin-like protein ligase binding pathways closely related to protein degradation were only involved in the M6-M12 group. Taken together, the genes regulated by DNA methylation were integrated with the biological processes of muscle development and protein degradation, and so on to influence the growth of postnatal muscle.

To further study the possible binding of TF in LMR and the regulatory network of those genes in muscle development-related pathways, a similar strategy was adopted to construct the TF-targets regulatory network (LMR network): TFs were first predicted by the motif of LMR, and the relationship with the DMR-regulated genes, which were found in TF database as target genes of the predicted TFs, were then visualized in a network. Many stage-specific TFs including *CTCF*, *STAT5A*, *ELK4*, and so on were found (Fig. 6c). To narrow down candidate genes, the targets in D3-M6 and D3-M12, which covered most pathways related to muscle development in Fig. 6b, were considered. Any TF found in LMR network was marked in red (Fig. 6c). Similarly, targets in muscle-related pathways were labeled in red (Fig. 6d) according to the GO enrichment results of the network genes which were observed in the muscle system process, striated muscle cell differentiation, skeletal muscle organ development, and muscle atrophy (Fig. 6e). In the network, *ENO1* is regulated by three kinds of TFs: *CTCF*, *ELK4*, and *MAZ* at the D3 stage and *CTCF* at the M3 stage. In the genome browser, there is a significant change in the DNA methylation level of *ENO1* in the anterior part of exon 10 during muscle development: D3, M3 methylation level is close to 0, while M6, M12 is about 50% (Fig. 6f and Supplementary Fig. 6a). The negative correlation of methylation and expression of *ENO1* has not reached a significant level yet (Rho: 0.8, *P* value: 0.3, Supplementary Fig. 6b and Supplementary Data 4).

### Interaction between DNA methylation and chromatin accessibility

**Fig. 7:**
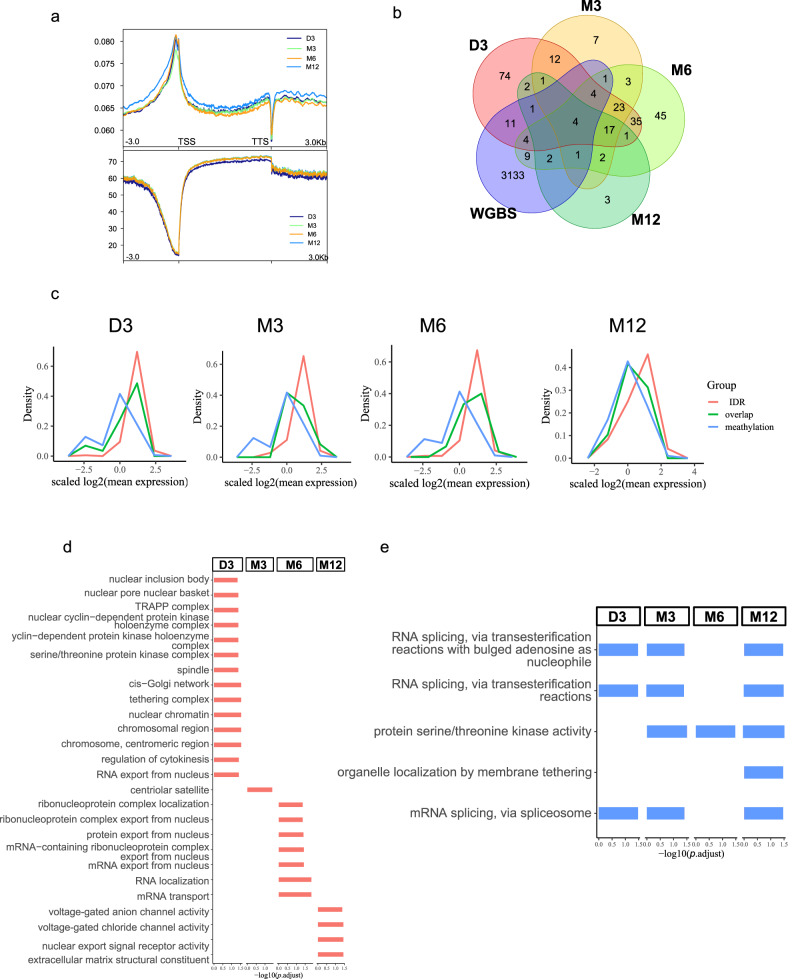
Interaction between DNA methylation and chromatin accessibility during postnatal muscle growth. **a** Genome-wide accessible signal (top) and mean methylation (bottom). TSS refers to the transcriptional start site, and TTS refers to the transcriptional terminal site. **b** Venn diagram of loci with IDR peaks and methylated sites on TSS ± 3 kb (kilobase). **c** Expression distribution of three types of genes: genes with IDR peaks only, genes with methylated sites only and those had both modifications on TSS ± 3 kb. **d** GO items for genes with IDR peaks only. **e** GO items for genes with methylated sites only. IDR irreproducible discovery rate.

To explore the interaction between DNA methylation and chromatin accessibility during skeletal muscle growth, TSS ± 3 kb was considered due to the high variation of IDR signals and average methylation near TSS across four stages (Fig. 7a). In the four stages (D3, M3, M6, M12), 24, 10, 25, and 8 loci with possible interaction effects were respectively identified by the intersection of genes with DAR or DNA methylation signals on TSS± 3 kb region, of which 14, 4, 10, and 2 genes were annotated (Fig. 7b).

**Fig. 8:**
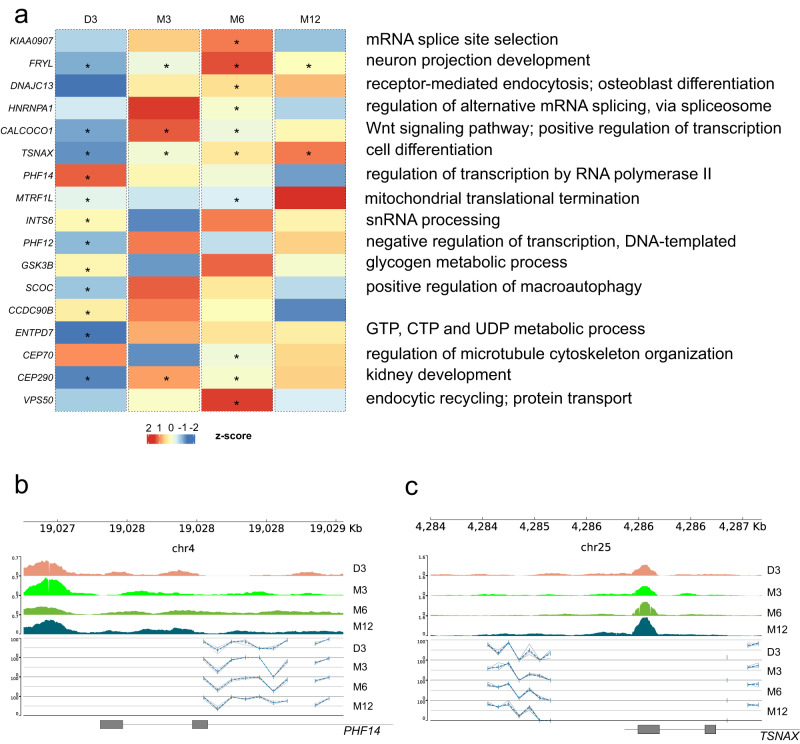
Candidates subjected to temporal and persistent interaction effects between DNA methylation and chromosome accessibility. **a** Expression heatmap of genes subjected to IDR and DNA methylation effects with biological processes list of corresponding genes, according to genecards.org, aside. “*” means when the interaction effect occurred. *PHF14* only had IDR peaks at D3 stages, while *TSNAX* had constant IDR peaks across four stages, as “*” indicated. The Genome browser displayed *PHF14* (**b**) and *TSNAX* (**c**). GCF_000298735.2 was used for genome annotation.

Those genes were displayed according to their gene expression in corresponding stages. For clarity, intersection (green line in Fig. 7c) refers to genes that were subjected to the effects of both DAR signals and DNA methylation, while IDR (orange line in Fig. 7c) and methylation (blue line in Fig. 7c) refer to which were independently affected by open chromatin signals (or IDR peaks) and DNA methylation, respectively. First of all, the expression pattern of genes of the intersection line (green line) fell somewhere between the IDR and DNA methylation sides, which means the expression of genes possibly mediated by IDR and DNA methylation was higher than genes affected by methylation but lower than genes affected by IDR (Fig. 7c) Then, in specific stages, the middle curve subjected to both epigenetic effects were closer to the IDR curve in D3 and M6 stage; while in the rest stages (M3 and M12 stages), it was closer to the methylation curve (Fig. 7c). This indicated that the effect size of IDR and DNA methylation on their host gene’s expression may differ as muscle grew. The IDR effect tended to have a stronger effect on their host genes than DNA methylation in D3 and M6, while weaker in M3 and M12. Interestingly, it was similar to the trend of IDR signals on CpG islands, which displayed a relatively high overlapping rate of IDR peak on CpG islands in D3 and M6 (Supplementary Fig. 2b). Interestingly, it was similar to the trend of IDR signals on CpG islands (Supplementary Fig. 2b). Secondly, these genes of IDR and DNA methylation sides were selected for GO enrichment analysis (Fig. 7d, e), and the genes mediated by both effects were annotated by the biological processes category of GO pathways (www.genecards.org, Fig. 8a). The genes regulated only by IDR peaks involved in translation activities and cytoskeleton organization (Fig. 7d), while the DNA methylation-regulated genes were mainly enriched in mRNA splicing and protein serine/threonine kinase activities (Fig. 7e). In the four stages, 17 genes were identified with the overlap of IDR and DNA methylation in TSS ± 3 kb, with “*” denoted in the corresponding stages of expression heatmap and noted with the pathways of biological process from GO database labeled aside. The first seven out of these (*CALCOCO1*, *DNAJC13*, *FRYL*, *HNRNPA1*, *KIAA0907*, *PHF14*, *TSNAX*) were found in DEGs. These genes are involved in mRNA alternative splicing, cell differentiation, metabolism as well as autophagy, etc. (Fig. 8a). Taking *PHF14* as an example, the expression of *PHF14* decreased since D3 and there was no IDR peak near TSS except D3 (an asterisk labeled in D3, Fig. 8a). Accordingly, in the D3 stage, there is a high open signal on the upstream of *PHF14*, so its transcription was the most activated (Fig. 8b). This might be because the weak open signal or no IDR peaks existed in the other stages, and the overall DNA methylation level in the adjacent regions was lifted.

The transcriptional level of *TSNAX* is another case, whose expression increased with the development of postnatal muscle. Consistent with the trend of transcription, there is an IDR peak near the TSS of *TSNAX* at all stages, and the open signal increases gradually. Among them, the open degree of D3 and M3 were comparable, but the methylation level in the upper reaches decreased in M3, which may promote the expression of *TSNAX* (Fig. 8c). In addition, the other genes can be found in Supplementary Fig. 7a-e.

### The effects of epigenetic modification on postnatal muscle fiber transformation

**Fig. 9:**
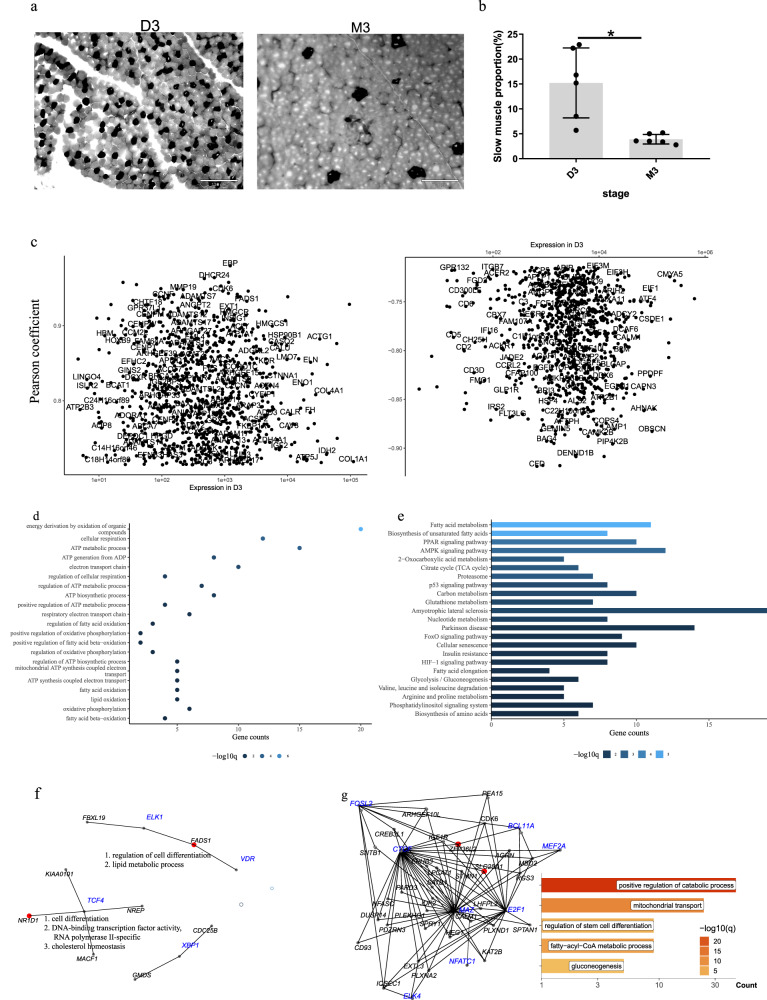
The effects of epigenetic modification on postnatal muscle fiber transformation. **a** Muscle fiber types distribution. Representative images of fiber types at D3 and M3 are shown. Scale bar: 130 µm. **b** Quantification of the proportion of the slow-twitch fibers at D3 and M3 (*n* = 6), *P* value = 0.01. **c** Genes with significant positive correlation and negative correlation with the proportion of the slow-twitch fibers. *x* and *y* axes, respectively represent gene expression in D3 and Spearman correlation efficiency (rho). **d** GO items for metabolic genes in (**c**). **e** KEGG items for metabolic genes in (**c**). **f** TF-targets networks of IDR (IDR network) based on metabolic genes and transcription factors in Fig. 4c. **g** TF-targets networks of LMR (LMR network) based on metabolic genes and transcription factors in Fig. 6c. Red targets are the metabolic genes in (**c**) and their GO items in (**d**) is in the lower right corner. Error bars represent mean values ± SD. IDR irreproducible discovery rate, LMR low methylation regions, SD standard deviation.

The composition of muscle fiber types is dynamic after birth. The proportion of slow-twitch muscle fibers decreased sharply from D3 to M3 by myosin ATPase staining. (Fig. 9a, b). A total of 1847 DEGs in D3-M3, with an absolute value of Rho greater than 0.7, were significantly related to the proportion of slow-twitch muscle fibers, and displayed in two dimensions: Spearman correlation coefficient (Rho) and their expression level at D3. (Fig. 9c). Retrieved from the Human Molecular Signatures Database (MSigDB), 229 out of 1847 genes involved in metabolism activities including energy derivation by oxidation of organic compounds, positive regulation of ATP metabolic process, ATP biosynthetic process, fatty acid oxidation, oxidative phosphorylation, etc. (Fig. 9d). Meanwhile, these genes were involved in the electron transport chain, cellular respiration, ATP metabolic process, and regulation of fatty acid oxidation, which was reflected in KEGG items such as the tricarboxylic acid cycle (TCA) cycle and fatty acid metabolism (Fig. 9e). In addition, KEGG enrichment also highlighted the biosynthesis of unsaturated fatty acids, glycolysis/gluconeogenesis, and PPAR, AMPK, p53, Foxo, and HIF signal pathways (Fig. 9e).

Next, we mapped the TF, inferred previously from IDR and LMR, and the above 229 metabolism-related genes to the hTFtarget database again to create TF regulatory networks involved in the transformation of fiber types. We further highlighted genes related to metabolism in the networks. *NR1D1* and *FADS1* were highlighted in the IDR network (Fig. 9f), which were both related to the regulation of cell differentiation. Besides, the LMR network found two important metabolism-related genes (*ZFP36L2* and *SLC25A1*), and both genes are regulated by *E2F1*, *FOSL2*, and *MAZ*. They are mainly involved in mitochondrial transport, stem cell differentiation regulation, acetyl-CoA metabolism, and gluconeogenesis (Fig. 9g). In addition, *MAZ* is D3-specific, while *E2F1* and *FOSL2* are M3-specific. Taken together, these results point out a hypothesis about the slow-twitch muscle fibers transformation: chromatin accessibility and DNA methylation might regulate the expression of the key genes via affecting TF binding at motif region and changes in oxidation metabolism pathways.

## Discussion

We performed a full comparison (a total of six comparison groups) among those four stages for RNA-seq, WGBS, and ATAC-seq data to ensure later analysis can be performed under the same comparison group. In terms of RNA-seq results, three important biological processes including protein synthesis, immune activities, and mitosis and their gene expression tendencies (T1–T3) were derived from the stratified DEGs through a combination of clustering methods, part of which also can be found on previous reports^25,26^. The main idea of this part of the analysis is to investigate the main transcription process of muscle postnatal growth in a robust way to avoid interference of false positives introduced by the case-control  comparison A non-linear unsupervised clustering method called a self-organizing map (SOM) was adopted to split DEGs into two groups (G1 and G2). In this step, SOM requires one-dimension input so we use the correlation between gene expression and the phenotype CSA. Next, we independently conducted a clustering method called Fuzzy C-Means Clustering on G1 and G2. In this step, we obtained self-confirmation results: the enrichment results showed the pathways in G1 can be validated in G2 with a similar expression pattern.

In this study, the threshold (Log2 fold change >0) was a less stringent cutoff than in other studies. On the one hand, we try to avoid excluding some translational factors with small-expression fluctuation yet highly efficient transcriptional activation and increase the candidate genes list for further screening. On the other hand, arbitrary filters can cause an unstable percentage of DEG in certain pathways. Furthermore, an increased DEG number will filter out pathways with a small number of genes annotated, which helps understand the main biological processes DEGs are involved. Therefore, we chose the *q*-value as the only threshold for DEGs.

As the ages, the ability of the muscle to regenerate is decreased, and the number of Muscle satellite cells declined^27^, which may be related to the prolonged infiltration of immune cells in T2 tendency. It is estimated that muscle satellite cells account for 30% of the total nucleus in early postnatal growth, while they account for only 2–7% of skeletal muscle in healthy adults and about 5% in older pigs. The study has shown that an increase of TNFα and IL6 levels in satellite cells or myofibril environment leads to skeletal muscle aging^28^. Moreover, some studies have shown that the continuous activation of NF-κB leads to telomere shortening of Muscle satellite cells under chronic injury, which has nothing to do with proliferation. It ultimately leads to the loss of Muscle satellite cells, and the failure of skeletal muscle regeneration^29^.

The core of muscle fiber hypertrophy is the net accumulation of cellular component proteins (e.g., myofibril, mitochondria, sarcoplasmic reticulum, cytoskeleton, etc.), which means that anabolism should exceed catabolism for a long time. Protein synthesis depends on the efficiency of ribosomal translation, and muscle nucleus density to provide a platform for mRNA transcription. There is a strong linear correlation reported in previous studies among the CSA of human muscle fibers, the number of nuclei per fiber, and ribosome content (RNA concentration)^3,30^. The current viewpoint^31^ is that: (1) the initial stage of muscle fiber hypertrophy induced by mechanical overload is not necessarily satellite cell-dependent. Instead, it can occur mainly through protein accumulation; (2) as the CSA of a single fiber and the size of a single nucleus domain enlarges, Muscle satellite cells mediated myonuclear addition may be essential for sustained hypertrophy.

Studies from the single-cell RNA-seq have confirmed that damaged muscle satellite cells activate cell differentiation and gradually increase ribosomal biogenesis and metabolic activities like glycolysis, tricarboxylic acid cycle (TCA cycle), and fatty acid oxidation. In addition, there are significant differences between primary myoblasts and muscle satellite cells^21^: both primary myoblasts and muscle satellite cells cluster enriched cell cycle-related pathways, but metabolism, protein balance, and translation activation were also found in the primary myoblasts cluster. Interestingly, pathways related to the cell cycle were also enriched in T1 tendency which features protein synthesis. This is a positive indication that genes in different trends (T1–T3) are well coordinated, not just within similar trends.

Chromatin accessibility is directly tied to gene transcriptional regulation. Particular attention was given to the role of enhancers and differential genes sharing the same direction of fold change between DAR and DEGs were selected. Most genes belonged to the T3 trend, and only a few genes were assigned to T1 and T2 trends such as in D3-M6. Some studies have reported genes shared reverse fold change and speculated that transcriptional suppressors may be involved^32^. Our study further found that the upregulated minority was closely related to muscle development.

To find the potential relationship between accessible regions and DEG, we map the TF inferred from IDR peaks and DEGs which shared the same directions of fold changes with DAR to the hTFtarget, an external TF-targets database, to construct the IDR network. Motif enrichment analysis found a series of transcription factors and their target genes: *FoxO1, IFRD1,* and *ARID5B* belonged to the T1 trend, while *ANXA6,ADORA2B* and *SMAD7* genes belonged to the T3 trend. Combined with the biological processes assigned to T1 and T3 trends, it also makes sense that these targets are essential for muscle development. Although most studies about SMAD7 focused on muscle differentiation, a study of systematic administration of rAAV6:SMAD7 delivery confirmed the hypertrophy of muscle mass body-wide on C57BL/6 mice^33^. *ARID5B* is involved in the formation of H3K9me2 demethylase. *ARID5B* knockout increases glucose uptake, metabolism, and oxygen consumption in skeletal muscle^34^. Besides, it also leads to the hindrance of cell differentiation and sarcomere defect in primary skeletal muscle^35^.

DNA methylation is generally considered a static epigenetic modification, but some studies have found that dynamic DNA methylation plays an important role in regulating muscle metabolism^36^. We calculated the mean methylation at four annotation sites mentioned in Method Annotation for ATAC-seq and WGBS part: the upstream region, the gene body, the downstream region, and the distal region. Then *t* test was performed among these four stages (a total of six comparison groups), and the *P* value was adjusted with the FDR method. Although there were no positive results (no significant comparison group was reported), it’s still a simple way to present the gradually increased methylation level along with postnatal muscle growth to avoid thousands of outliers. In our study, the average methylation of DMR climbed largely (40-60%) since D3, at a decreasing speed. Interestingly, the GO terms of many DMR groups reached a consensus in pathways related to muscle development, such as muscle system process and actin filament organization. By further combining with DEGs, those genes affected by DNA methylation are enriched into more muscle-related pathways in the D3-M6 and D3-M12 groups.

As for the classification types based on methylation types, LMR and UMR are potentially important regulatory regions, due to their low methylation characteristics; We mainly focused on the potential regulatory relationship between LMR (we merged it with UMR, see methods) and the intersection of genes of DMR and DEGs. Therefore, we infer motifs (or transcription factors) from the identified LMR. Then we narrowed down the intersection genes of DMR and DEGs according to their enrichment results and connected these genes with previously mentioned motifs inferred from LMR.

In LMR network, *ENO1* may be involved in glycolysis, plasminogen activation, and transcriptional inhibition. It has been reported that human α, β enolase is a marker of early myogenic differentiation^37,38^. The expression level of total enolase is closely related to the maintenance of muscle fibers, and knocking down *ENO1* reduces the fusion efficiency of myoblasts^37,39^. Other studies have also found that Wnt5a, Wnt9a, and TGFβ1, as key nodes, regulated the functions of satellite cells^40^.

Our study hypothesized that the location changes of IDR and LMR may contribute to spatial-specific, temporal-specific, or cell-type-specific regulation of TFs. Several studies^41,42^ used ATAC-seq to study the specific regulation of transcription factors, but DNA methylation was not considered. At present, the function of some TFs found in our study, such as *VDR*^43^, *MEF2*, *FOSL2*, *JUND*^42^, *TCF4*, *TEAD3*^44^, and *MYF5* have been reported. Among them, members of the myogenic regulatory factor family of transcription factors *MYOD*, *MYF5*, *MRF4*, and *Myogenin* (*MYOG*) are the key regulators of muscle gene expression in vertebrates during early and adult myogenesis^45–47^. It is worth noting that these enhancers activate transcription through direct contact with the promoter of target genes, via the CpG-bound TF NRF1 and the formation of CTCF-anchored chromatin loops in a muscle fiber-specific manner^48^. Besides, histone H4 and E2F2 bind to the -216/-28 region and play important roles in *SIX1* methylation regulation^49^.

The TSS ± 3 kb region is suitable for studying the interaction between DNA methylation and chromatin accessibility because highly accessible chromatin and hypomethylated cytosine were found to co-exist. The interaction is already implied in the DMR heatmap in which no linear correlation was observed between the DNA methylation and expression of DEGs. According to the expression distribution of the two epigenetic modified genes, three parts were identified for each stage: genes affected independently by IDR or DNA methylation and genes under both effects. We reported that the bias of expression distribution with interaction effect was similar to that of IDR proportion in CpG island. This not only further supported the interaction but also suggested that CpG island may be the place to link the two of epigenetic modification. Aligned with this finding, the enrichment results of intersect genes of DMG and DEGs were found 12 and 7 significant pathways related to muscle for D3-M6 and D3-M12 groups, respectively. A total of 17 genes with possible interaction effects were obtained, among which *CALCOCO1*, *DNAJC13*, *FRYL*, *HNRNPA1*, *KIAA0907*, *PHF14*, and *TSNAX* were DEG. *PHF14* (PHD Finger Protein 14) is a hypoxia-sensitive surface modification regulator, which encourages cell cycle progression and protein synthesis. Hypoxia-induced down-regulation of *PHF14* inhibits the transition from the G1 to the S phase of mitosis and compromises protein synthesis by inhibiting the AKT-mTOR-4E-BP1/pS6K pathway. In our result, the expression of *PHF14* was the highest in the D3 stage, followed by M3-M6. This may be related to satellite cell proliferation and muscle protein synthesis^50^. *TSNAX* binds to Translin as an endonuclease activated by RNA-induced silencing complex. Consistent with the results, *TSNAX* was highly expressed in the chest muscle, leg muscle, and heart of ducks, as muscle aged^51^.

The body’s musculature is composed of a variety of muscle groups, which enable it to perform specific motor activities. Different muscle groups contain heterogeneous muscle fibers with distinct biochemical, contractile, and metabolic properties^52^. Muscle fibers are classified into slow-twitch and fast-twitch fibers based on contractile performance and the expression of specific isoforms of myosin heavy chain. Slow-twitch muscle fibers, rich in myoglobin and oxidative enzymes and specialized for more continuous activity, and fast-twitch characterized by glycolytic metabolism and specialized for phasic activity^53^.

Different types of muscle fibers feature divergent metabolic phenotypes, which contribute to meat quality or muscle performance. With the development of muscle, the proportion of slow-twitch muscle fibers was decreased. The D3 and M3 stages were investigated to analyze the effect of chromatin accessibility on slow-switch muscle fiber transformation. IDR-network analysis showed *NR1D1 and FADS1 were * key candidates.  *NR1D1* is highly expressed in oxidative skeletal muscle and plays a role in mitochondrial biogenesis, oxidative function^54^ and calcium homeostasis^55^. In addition, the LMR network found the metabolism-related gene *ZFP36L2*, a member of the *ZFP36* family, exists in both IDR network and LMR network. Its analog *ZFP36L1* is also in LMR network. *ZFP36* regulates the degradation rate of mRNA and the myogenic process by binding to the AU-rich sequence in 3’UTR of mRNA^56^.

**Fig. 10:**
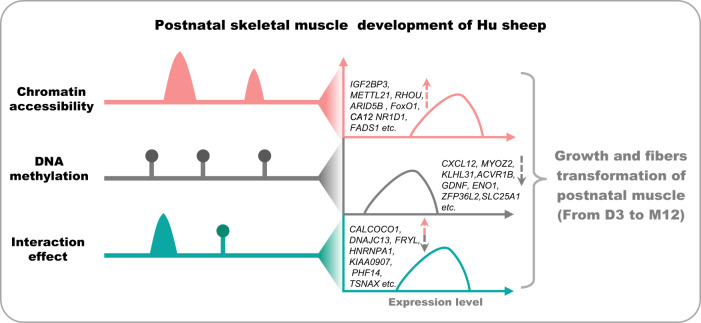
Summary of potential regulatory patterns of DNA 5mC and accessible chromatin regions on gene expression. On the one hand, the expression of the genes influencing growth and fiber transformation during postnatal skeletal muscle in Hu sheep can be independently either upregulated by chromatin accessibility or downregulated by DNA methylation at specific stages. For example, *ARID5B* may be upregulated by *XBP1* in M3 than D3, due to increased accessible signal on the UTR of one of its transcripts. On the other hand, the interaction of chromatin accessibility and DNA methylation maintained a moderate level of the genes related to skeletal muscle growth. Some genes, like *CALCOCO1* and *PHF14*, were possibly mediated by both 5mC and accessible chromatin in our dataset.

In summary, the combined ATAC-seq and WGBS with RNA-seq was applied to discover the epigenetic dynamics, genome-wide DNA methylation and chromatin accessibility, and its regulations during postnatal muscle development of Hu sheep. In four stages (from D3 to M12), all DEGs were stratified into two groups according to the correlation with CSA, and the two group genes were further assigned into three distinct tendencies (T1-T3), which were mainly enriched in the regulation of protein synthesis, immune activities, cell division and so on. IDR network from chromatin accessibility-regulated genes showed the *ARID5B*, *SMAD7, FoxO1, etc* were identified in muscle organ development pathways. Global genome DNA methylation forms were mainly the contexts of methylated cytosine, and the genes regulated by DNA methylation were integrated with the biological processes of muscle development, protein degradation, and so on. The genes such as *ENO1*, *MYOG*, *IGF1R*, etc. were observed in muscle system process, skeletal muscle organ development, muscle atrophy, and so on by LMR-network analysis. the interaction between DNA methylation and chromatin accessibility at TSS ± 3 kb region showed 17 genes (*KIAA0907*, *PHF14*, *TSNAX*, etc.) were identified with interaction effects which were involved in mRNA alternative splicing, cell differentiation, autophagy, etc. the metabolism-related genes (*NR1D1*, *FADS1*, *ZFP36L2*, and *SLC25A1*) were involved in the transformation of slow-twitch muscle fibers using IDR-network and LMR-network analysis, which were enriched in the acetyl-CoA metabolism, fatty acid oxidation, gluconeogenesis and so on. Taken together, the genome-wide DNA methylation and chromatin accessibility were involved in regulating the growth and muscle fiber type transformation of postnatal skeletal muscle (Fig. 10).

## Methods

### Ethics statement

All experimental animal protocols were approved by the Animal Care and Use Committee at China Agricultural University (Aw03602202-1-1).

### Animals and muscle histology

Six Hu sheep (three males and three females) of the same age were normally developed, vital, and without visible defects. At the four developmental stages (D3, M3, M6, and M12), the quadriceps femoris tissues of these six Hu sheep were obtained by local operation. To avoid the interference experiment of muscle regeneration and repair-related gene expression, we increase the time interval of sampling at the same position as much as possible. The quadriceps femoris tissues of the left leg were operated at D3 and M6, while the right leg was handled at M3 and M12.

The quadriceps femoris tissues of six Hu sheep were frozen rapidly with liquid nitrogen. A total of 6 µm sections were cut using a low-temperature microtome, affixed to a slide, and stored at -80 °C until dyed. For the calculation of fiber cross-sectional area, Wheat Germ Agglutinin (WGA) with fluorescent-conjugated (AlexaFluor488) (W11261, Invitrogen, USA,) was used to stain the extracellular matrix. The working concentration of WGA was 50 ng/ml. Remove the slices and place them at 37 °C for 2 min. The tissues were covered with WGA working solution and incubated at 37 °C for 30 min. Wash with PBS three times, 5 min each time. Identification of fast and slow-twitch muscle types by ATPase staining^57^, The sections were placed in an acidic pre-incubation solution and incubated at room temperature for 30 min. The slides were taken out and washed with double distilled water for 1-2 min. The water droplets on the surface of the slices were dried as much as possible (keeping the tissue sample wet), put into a dye vat containing ATP incubation solution, and stayed overnight at 4 °C. The slices were quickly washed once in 1% CaCl_2_ solution, replaced with 1 % CaCl_2_ solution, and incubated for 3 min. The slices were incubated with 2% CoCl_2_ solution for 3 min. Tap water washes quickly (washing speed is fast, the sample will not fall off), and deionized water washes for 2 min. 200 µl 1% ammonium sulfide was dropped on tissue sections for 2 min. Washed with tap water and deionized water for 2 min. The black staining of slow-twitch muscle fibers and gray staining of fast-twitch muscle fibers.

### Measurement of CSA and count of slow-twitch muscle proportion

Original images were captured at ×20 objective magnification using an ECHO microscope (American).

We used Python^58^ and OpenCV (https://docs.opencv.org/4.5.3/index.html) and^59^ to compute the CSA of muscle fibers. First, the captured RGB image is preprocessed by gray transformation. Then, the noise and background were filtered out in the gray image by using a Gaussian filter and image binarization. Subsequently, the morphological operation was implemented to select candidate muscle fibers. The threshold for the area is ranging from 1400 pixels to 80,000 pixels, and the circularity of the selected region is ranging from 0.2 to 1.0. Finally, to compute the CSA of muscle fibers more accurately, we used morphological operation again to remove the adhered muscle fibers and fill up the remained muscle fibers. The calculation of slow-twitch muscle proportion using Image-Pro Plus 6.0 software.

### Transcriptome sequencing (RNA-seq)

The total RNA was extracted from the quadriceps femoris using a Tissue RNA Kit according to the manufacturer’s instructions (Omega Bio-tek, USA). The RNA concentration was measured using an Agilent 2100 Bioanalyzer and Agilent RNA 6000 Nano Kit, and the RNA purity was verified by a NanoDrop 2000 micro-spectrophotometer. The integrity of RNA was analyzed by Agilent 2100. Libraries of six Hu sheep were prepared and sequenced on the Illumina platform for high-throughput sequencing with read PE150 (Frasergen Bioinformatics, Wuhan, China).

### Targeting accessible chromatin with high-throughput sequencing (ATAC-seq)

In this experiment, ATAC-seq was completed in Frasergen Bioinformatics, Wuhan, China. Frozen tissues from three Hu sheep were homogenized into cell suspensions with cold PBS buffer. Lysis buffers were added to the cell suspensions and incubated for 10 min at 4 °C on the rotation mixer. Cell suspensions were filtered with a 40-µm cell strainer and then washed with cold PBS buffer once time. Approximately, 50,000 nucleis were added to the transposition reaction solution to perform tagmentation. Tn5 transposed DNA was purified by AMPure DNA magnetic beads and PCR amplification. Qubit2.0 was used for preliminary quantification. The library was diluted to 1 ng/μl, and Agilent 2100 was used to measure the fragment size of the library. The effective concentration of the library was further determined by Q-PCR (>2 nM) to ensure the quality of the library. The final qualified library was sequenced on the Illumina Nova-seq platform (San Diego, CA, USA) with PE150 mode.

### Whole-genome bisulfite sequencing (WGBS)

In this experiment, WGBS was completed in Frasergen Bioinformatics, Wuhan, China. Extraction of genomic DNA from the quadriceps femoris of three Hu sheep. In total, 2 μg of genomic DNA spiked with 5 ng unmethylated Lambda DNA (Promega, Madison, WI, USA) was fragmented by sonication to a mean size of ~200-400 bp. 500 ng of purified fragmented was end-repaired, 5’-phosphorylated, 3’-dA-tailed and then ligated to 5-methylcytosine-modified adapters. The bisulfite conversion was carried out using the ZYMO EZ DNA Methylation-Gold Kit (Zymo Research, Irvine, CA, USA) and amplified via PCR with ten cycles using KAPA HiFi HotStart Uracil+ ReadyMix (Kapa Biosystems, Wilmington, MA, USA) and Illumina 8-bp index primers. The constructed WGBS libraries were then analyzed by Agilent 2100 Bioanalyzer and finally sequenced on the Illumina platform. The sodium bisulfite non-conversion rate was calculated as the percentage of cytosines sequenced at cytosine reference positions in the lambda genome.

### Sequencing data processing

Except for the RNA-seq (six replicates for each stage), ATAC-seq (three replicates for each stage), and WGBS data (three replicates for each stage), we also download WGS data to identify methylation types and ChIP-seq data to visualize the activation signals (H3K4me3, H3K27ac, etc.) with accessible signals. Associated metadata can be seen in Supplementary Data 5. The analysis software and associated datasets used are listed in Supplementary Table 1.

Trim_galora are used for filtering, including the removal of adapters and low-quality reads. The filtered data is called clean data. Clean data was aligned with the sheep reference genome by different aligners.

For RNA-seq data, a total of 242 G sequencing data was obtained, with an average Q30 of 92%. Hisat2^60^ 2.2.1 was used for alignment. Then BAM files were sorted by samtools^61^ 1.9 and quantified by featureCounts^62^ of subread 2.0.1. the PCA and hierarchical clustering methods were adopted to detect outliers. As a result, a total of 21 samples were preserved for downstream analysis, while three outliers (respectively in D3, M6, and M12) were excluded. The DEGs are obtained by DESeq2^63^ 1.26.0, with criteria as |Log2FoldChange | > 0 and *q*-value < 0.05.

For ATAC-seq and ChIP-seq, clean data was aligned by Bowtie2^64^ 2.35.1, and Samamba^65^ 0.6.6 was used to remove PCR duplicates. Use MACS2^66^ 2.1.0 to obtain the signal peak of the open area, with the effective genome size set as 2.20E + 06. For ATAC-seq data, parameter was set as --keep-dup=all --cutoff-analysis -g 2.20E + 06 -B --SPMR --nomodel --shift -75 --extsize 150; Following fetal ChIP-seq samples were download: H3K4me3 samples (SRR5070525-SRR5070530), H3K27ac samples (SRR5070519-SRR5070524) and nucleosomal DNA (SRR5070531). The parameter was set as --keep-dup all --nomodel -- extsize 100 -g 2.20E + 06 in MACS2. To get the consistent accessible peaks, use the IDR^67^ (Irreproducible Discovery Rate) method within the repeated samples, and use BEDTools^68^ intersect -wo to get the intersection.

For WGBS data, Bismark^69^ 0.23.0 software which calls Bowtie2 aligns the clean data of WGBS data to the reference genome, uses deduplicate_bismark to remove duplicate reads, and uses bismark_methylation extractor function to quantify methylation. A bulk DNA sample was used, so the methylation level of the cytosine base ranges from 0 to 100.

Besides, WGS (whole-genome sequence) data were used for better methylation types identification, which requires the location of CpG island and SNP to exclude CpG islands overlapped with SNP and find regions with low methylation levels more precisely. To get more precise SNP data, we used the intersection results of SNPs identified from the public database and our WGBS data. 6 WGS samples^70^ (one ewe SRR10821772 and five rams SRR11657579-SRR11657583) were downloaded from the NCBI SRA database and associated metadata can be seen in Supplementary Data 5. To note, the only consideration about selection is including both male and female HU sheep. However, its proportion was not yet under serious consideration.

Following SNP calling processes were conducted: (1) fastqc for data quality control; (2) BWA MEME 2.0 (doi.org/10.48550/arXiv.1303.3997) for alignment; (3) Samtools for sorting, indexing, and using Samamba to remove PCR duplications; 4) using HaplotypeCaller, CombineGVCFs, SelectVariants, VariantFiltration built-in GATK4^71^ 4.1.8 to screen SNP mutation types and get high-quality mutations (QUAL > 30). To better adapt to the data of this study, SNPs were also called based on WGBS data by Biscuit 0.3.16 (github.com/huishenlab/biscuit). Intersected loci were used for downstream analysis.

### Annotation for ATAC-seq and WGBS

ChIPseeker^72^ 1.26.2 can annotate a BED file with gene name, distance from TSS, genome regions, etc. ChIPseeker annotates in the following order: promoter,5’UTR, 3’UTR, exon, intron, downstream, distal intergenic. It is important to note that we defined promoter as 3 kb upstream of TSS in ChIPseeker to classify genomic region into four categories: upstream region (including promoter (TSS-3kb) and 5 ‘UTR in the original classification), gene body (including Exon and Intron above), downstream region (Downstream and 3’ UTR above), and distal region (distal intergenic).

### Clustering analysis of RNA-seq data

For each DEG, the Spearman rank correlation coefficient (rho) was first calculated between expression and CSA. DEGs were ranked by the rho value and clustered by a self-organizing map (SOM) implemented in the Kohonen^73^ 3.0.10 R package. SOM clustering is an unsupervised clustering algorithm, which is often used with hierarchical clustering. Then, we use the built-in function hclust to assign DEGs into two groups. Finally, time expression patterns were identified for each group using the Mfuzz^74^ 2.46.0 package.

### Overrepresentation analysis

ClusterProfiler^75^ 3.14.3 was used for overrepresentation analysis within GO and KEGG databases. *P* value was corrected by the false discovering rate (FDR) method (*P*.adjust), the significant threshold was 0.05, and similar enrichment pathways (default parameters) were removed by semantic similarity.

### Methylation types identification

As mentioned above, MethylSeekR^76^ 1.26.0 software requires the location of the CpG island and SNP to classify the methylation level of the whole genome. The reason to exclude the CpG islands overlapped with SNP is simply due to the difference in genome background between real samples and the reference genome.

Partially methylated regions (PMD), low methylated regions (LMR), and unmethylated regions (UMR) were obtained in turn: PMD needs to be masked before the segmentation of low methylation regions (LMR and UMR); HMD was the rest of the regions with high methylation levels.

Originally identified UMR and LMR were merged and named with LMR, because the range of methylation level of LMR and UMR is highly overlapped (Wilcoxon test: *P* value = 0.69).

### Differential accessed regions identification

The results of the MACS2 can be used for DAG identification with DiffBind^77^ 2.14.0, which calls DEseq2 for difference analysis. The least qualified sample was selected as the control bam to reduce the noise produced by liquid nitrogen (N2) Cryopreservation. The criteria for DAR are: adjusted *P* value by FDR < 0.1. Note that the Diffbind uses DEseq2 to calculate log2 normalized read counts (average within groups). Genes with DAR are called differential accessible genes (DAG). All the parameter settings use the default parameters of the software. PCA plot was drawn by DiffBind dba. plotPCA functions.

### Differentially methylated regions identification

DMR refers to some fragments that show different methylation patterns in different samples. Because DSS^78^ 2.38.0 requires the methylation level of input files to be a percentage, the awk software is used to convert the cov file exported by Bismark. Use the default parameter of DSS to get DMR. Genes with DMR annotation are called differentially methylated genes (DMG).

### TF binding motifs analysis

The findMotifsGenome.pl of HOMER^79^ 4.11 was used to analyze the potential transcription factor binding sites of given sequences. To reduce the false positive, the de novo algorithm was adopted, and the significance threshold was 10e-13.

### TF network construction

To find the potential relationship between potential regulatory regions and DEGs, we first inferred the potential binding site of TF from IDR which ensures high-confidence open chromatin peaks, and from LMR which features low methylated cytosine coverage, respectively. Both IDR and LMR are potential regulatory regions that were identified through different perspectives.

Then we selected candidates starting from DEGs. We chose DEGs which shared the same directions of fold changes with host genes of DAR as candidates for IDR network; while we chose the intersection of DEGs and host genes of DMR. To narrow down the candidate genes of LMR network further, we selected specifically the targets in D3-M6 and D3-M12, which covered most pathways related to muscle development in Fig. 3g.

Next, the TF and candidates were mapped to the hTFtarget^80^, an external TF-targets database, to connect both components and construct the IDR network and LMR network.

As for the regulatory network of the proportion of slow-twitch muscle fibers, we replaced the target genes of the above TF networks with the DEGs which were significantly correlated with SFP and also reported to do with metabolism in MSigDB.

### Genome browser

By summarizing the signal peak, methylation loci, gene structure, and differential results, the pyGenomeTracks^81^ 3.6 package is used for visualization on the genome browser. The genome annotation file (GCF_000298735.2) was used.

### Statistics and reproducibility

The CSA and values are reported as means ± SD (*n* = 6, animals). The differences of the CSA were analyzed by one-way ANOVA with SPSS. The differences of the slow-twitch muscle proportion were analyzed by independent-sample *t* test with SPSS. The statistical significance was defined at *P* value < 0.05. Multiple test correction is adjusted by the FDR method if no extra claims. Spearman Correlation coefficient (Rho) was adopted in calculating phenotype-related genes (*P* value < 0.05, if no extra claims).

### Reporting summary

Further information on research design is available in the Nature Portfolio Reporting Summary linked to this article.

### Supplementary information

Supplementary Information

Description of additional supplementary files

Supplementary Data 1-5

Reporting Summary

## Data Availability

Transcriptomic data were uploaded to the GeneBank database in NCBI Data Center under BioProject accession numbers PRJNA947918 (Registration date: 23 March 2023)^82^ and PRJNA1019899. ATAC-seq and WGBS data were deposited in the GeneBank under the project PRJNA1018291. ChIP-seq data are available in the NCBI SRA repository with the identifier: H3K4me3 samples (SRR5070525-SRR5070530), H3K27ac samples (SRR5070519-SRR5070524), and nucleosomal DNA (SRR5070531). WGS data are available in the NCBI SRA repository with the identifiers: SRR10821772 and SRR11657579-SRR11657583. Source data underlying figures are provided in Supplementary Data 1. All other data are available from the lead contact upon request.

## Code availability

The analysis methods and software used in this article are open source and listed in Supplementary Table 1. The code is available at the Zenodo repository (zenodo.org/record/8415716)^83^.
