# Supplementary material for: Genome-wide epigenetic dynamics during postnatal skeletal muscle growth in Hu sheep
Source: Commun Biol. 2023 Oct 23;6:1077. doi: 10.1038/s42003-023-05439-0 (PMC10593826; doi:10.1038/s42003-023-05439-0)
Supplement: Supplementary file 1 — Supplementary Information [file 42003_2023_5439_MOESM1_ESM.pdf]

## Supplementary Table 1

## Key resources table

| RESOURCE | SOURCE | IDENTIFIER |
|---|---|---|
| Deposited data | | |
| Sheep reference genome Oar_v4.0 (Oar_v4.0) | NCBI | https://www.ncbi.nlm.nih.gov/assembly/GCF_000298735.2 |
| WGS **(whole genome sequence)** data | NCBI SRA | SRR10821772, SRR11657579-SRR11657583 |
| ChIP-seq data (H3K4me3) | NCBI SRA | SRR5070525-SRR5070530 |
| ChIP-seq data (H3K27ac) | NCBI SRA | SRR5070519-SRR5070524 |
| ChIP-seq data (nucleosomal DNA) | NCBI SRA | SRR5070531 |
| TF database hTFtarget | Zhang,Q. et al, 2020 | http://bioinfo.life.hust.edu.cn/hTFtarget#!/download |
| Software and algorithms | | |
| Trim_galora 0.6.6 | the Babraham Institute | https://www.bioinformatics.babraham.ac.uk/projects/trim_galore/ |
| Hisat2 2.2.1 | Danecek, Petr et al., 2019 | http://daehwankimlab.github.io/hisat2/ |
| Samtools 1.9 | Danecek, Petr et al., 2021 | https://www.htslib.org/ |
| featureCounts subread 2.0.1 | Liao Y, Smyth GK and Shi W., 2014 | http://subread.sourceforge.net/ |
| DESeq2 1.26.0 | Love, Michael I., Wolfgang Huber, and Simon Anders. 2014 | https://bioconductor.org/packages/release/bioc/html/DESeq2.html |
| Bowtie2 2.35.1 | Langmead and Salzberg, 2012 | https://github.com/BenLangmead/bowtie2 |
| Samamba 0.6.6 | Artem Tarasov et al.,2015 | https://github.com/biod/sambamba |
| MACS2 2.1.0 | Zhang, Y., Liu, T., Meyer, C.A. et al., 2008 | https://github.com/macs3-project/MACS |
| BEDTools 2.29.2 | the Quinlan laboratory at the University of Utah | https://bedtools.readthedocs.io/en/latest/index.html |
| Bismark 0.23.0 | the Babraham Institute | https://github.com/FelixKrueger/Bismark |
| GATK4 4.1.8 | the Broad Institute | https://gatk.broadinstitute.org/hc/en-us |
| Biscuit 0.3.16 | the Van Andel Institute | https://huishenlab.github.io/biscuit/ |

| | | |
|---|---|---|
| ClusterProfiler 3.14.3 | G Yu, et al., 2008 | https://bioconductor.org/packages/release/bioc/html/clusterProfiler.html |
| ChIPseeker 1.26.2 | G Yu, et al., 2015 | https://bioconductor.org/packages/release/bioc/html/ChIPseeker.html |
| Kohonen 3.0.10 | Wehrens R, Kruisselbrink J, 2018 | https://cran.r-project.org/web/packages/kohonen/index.html |
| Mfuzz 2.46.0 | Matthias E. Futachik and Bronwyn Carlisle, 2005 | http://mfuzz.sysbiolab.eu/ |
| MethylSeekR 1.26.0 | Lukas Burger, et al, 2013 | https://bioconductor.riken.jp/packages/3.10/bioc/html/MethylSeekR.html |
| DiffBind 2.14.0 | Cancer Research UK's Cambridge Research Institute | https://bioconductor.riken.jp/packages/release/bioc/html/DiffBind.html |
| DSS 2.38.0 | Feng, Conneely, and Wu (2014) | https://bioconductor.riken.jp/packages/release/bioc/html/DSS.html |
| HOMER | Christopher Benner, University of California, San Diego | http://homer.ucsd.edu/homer/motif/ |
| pyGenomeTracks 3.6 | Fidel Ramírez, et al, 2018 | https://github.com/deeptools/pyGenomeTracks |
| IDR 2.0.4.2 | Li, Brown, Huang, and Bickel, 2011 | https://github.com/kundajelab/idr |

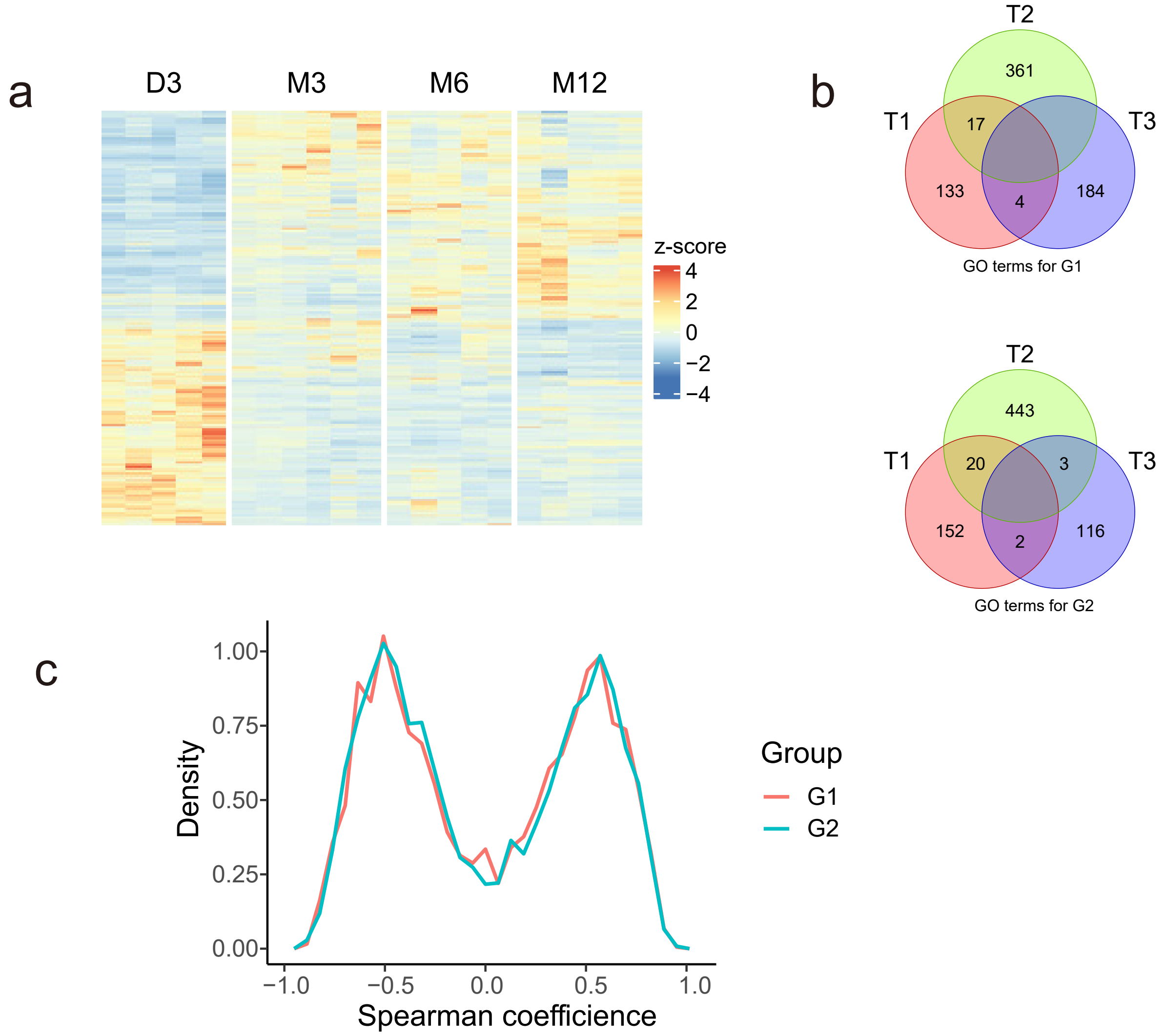


Supplementary Figure 1. **Difference among DEG groups base on expression cluster results** (a) Expression heatmap of DEG across four stages. (b) Venn diagram of GO terms of three tendencies (T1-T3) for G1 and G2 group, respectively. (c) Distribution of spearman correlation coefficient for G1(red line) and G2 groups (blue line). DEG: differntial expressed genes; GO: gene ontology.

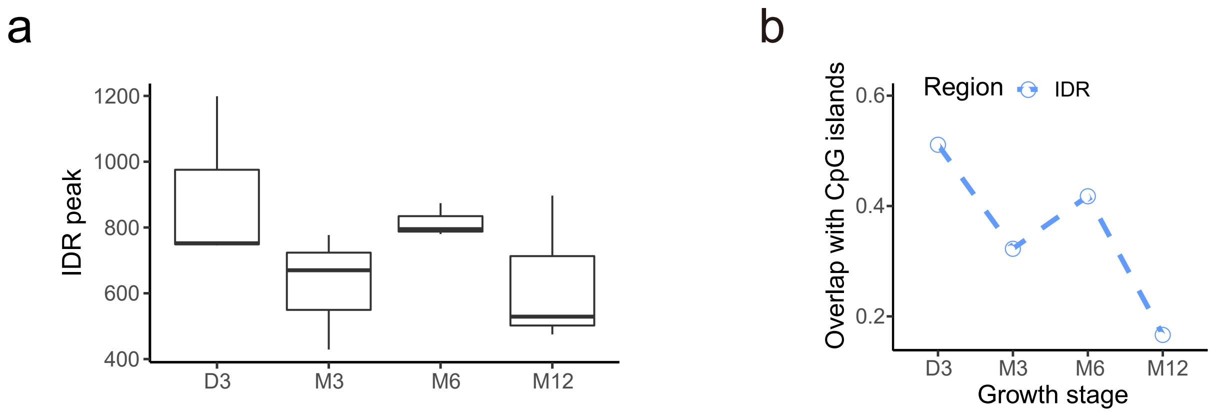


Supplementary Figure 2. **IDR peaks and its proportion overlapped with CpG islands** (a) Box plot summarizing number of IDR peaks across four stages. Boxes represent 25th to 75th percentile and whiskers represent minimum-maximum. Horizontal lines within boxes represent the median. (b) Line plot exhibiting number of IDR peaks located on CpG islands across four stages. IDR: irreproducibility discovery rate.

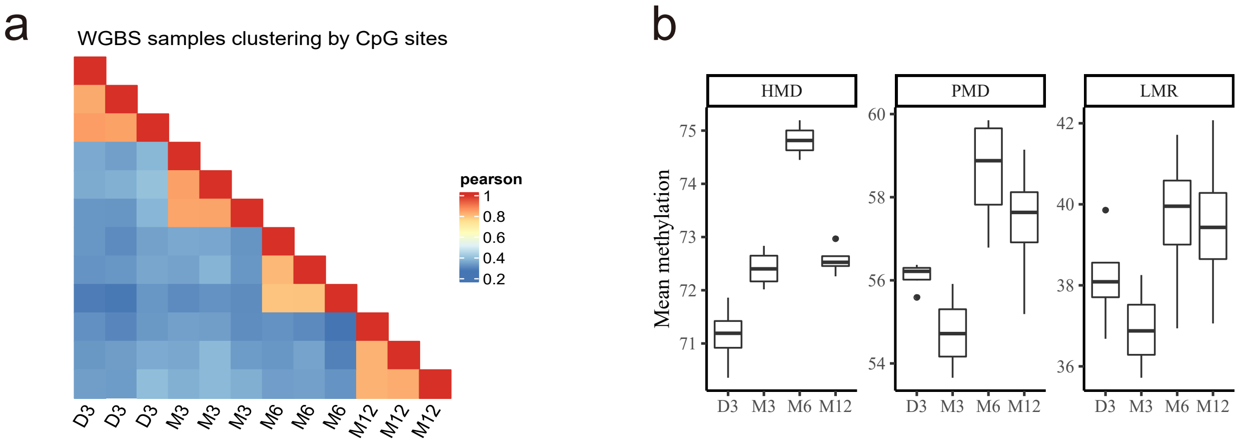


Supplementary Figure 3. **Overview of sample correlation based on methylation and methylation type**. (a) Heatmap of Pearson's correlation coefficient based on methylations of CpG sites. (b) Box plot summarizing mean methylation of HMD, PMD and LMR across four stages. Boxes represent 25th to 75th percentile and whiskers represent minimum-maximum. Horizontal lines within boxes represent the median. HMD: high methylated domains; PMD: partially methylated domains; LMR: low methylated regions.

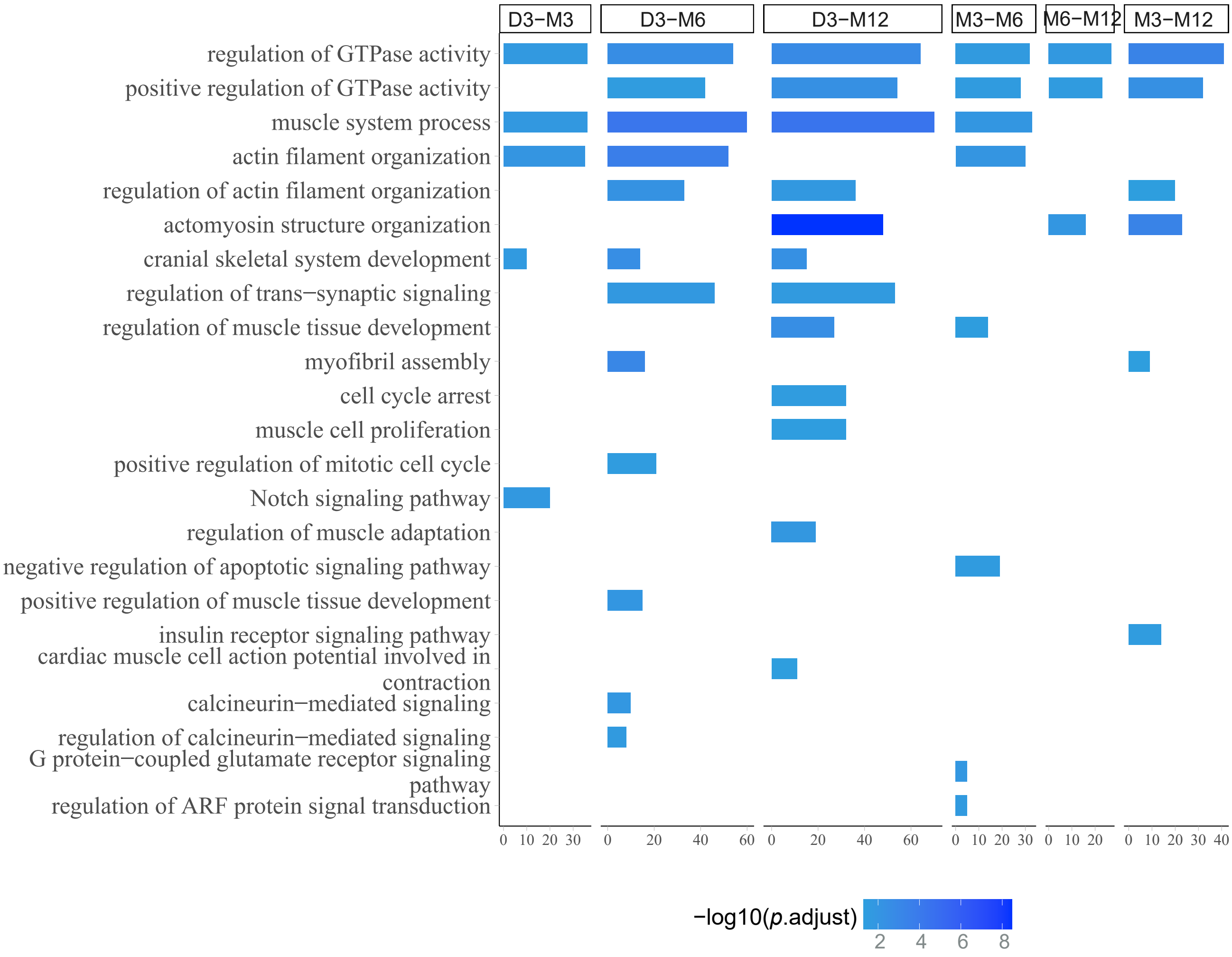


Supplementary Figure 4. **Overview of functional annotations of DMR**. GO items of DMR for all six comparison groups. DMR: differentially methylated regions. GO: gene ontology

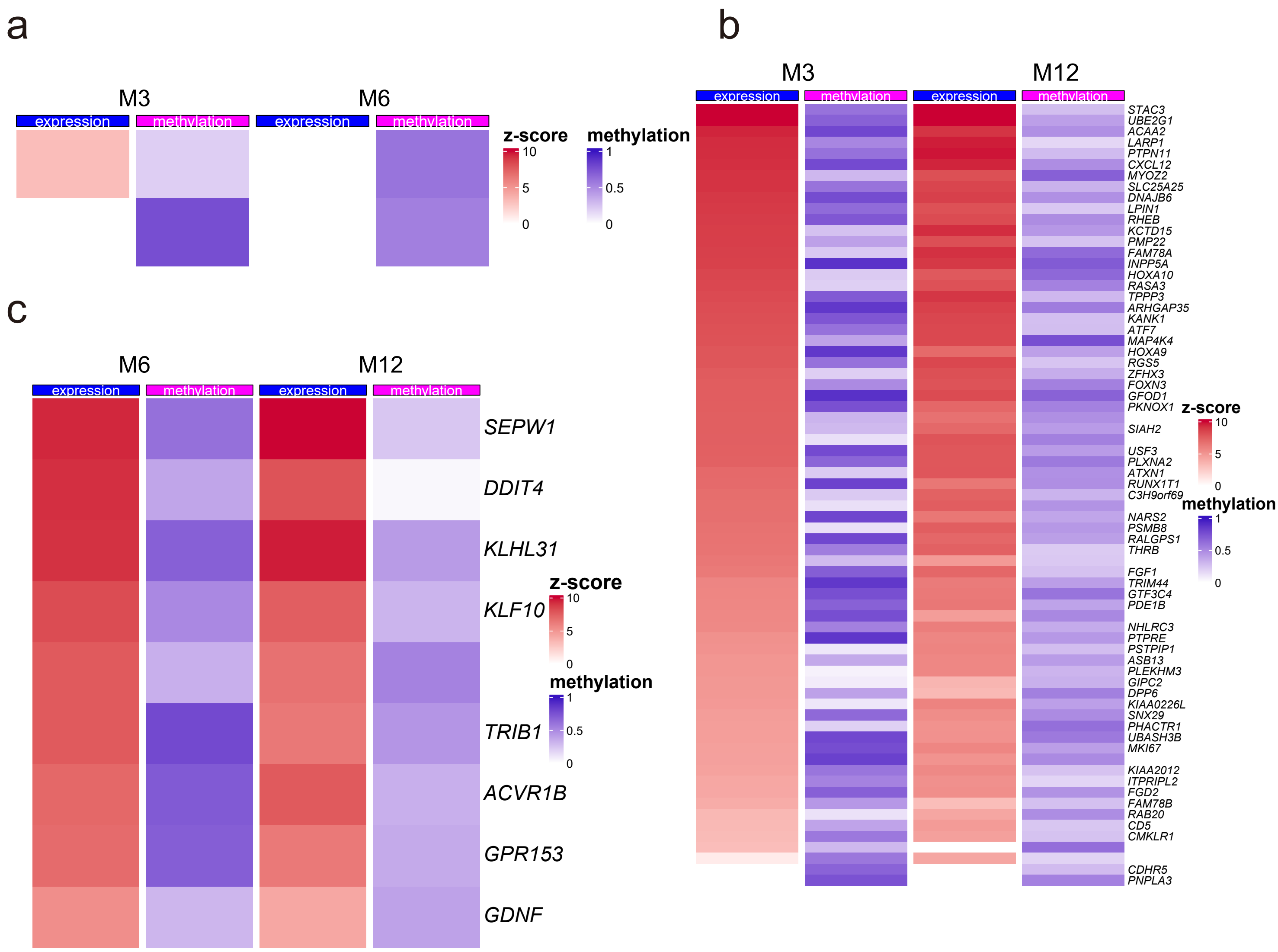


Supplementary Figure 5. **Methylation and transcription profile of DEG with DMR annotations**. Heatmap for gene expression (red -blue bar, red indicates high expression while blue means low expression) and their methylation at DMR (purple bar, the deeper purple, the higher DNA methylation) for M3-M6 (a), M3-M12 (b) and M6-M12 (c). DEG: differentially expresseded genes;DMR: differentially methylated regions.

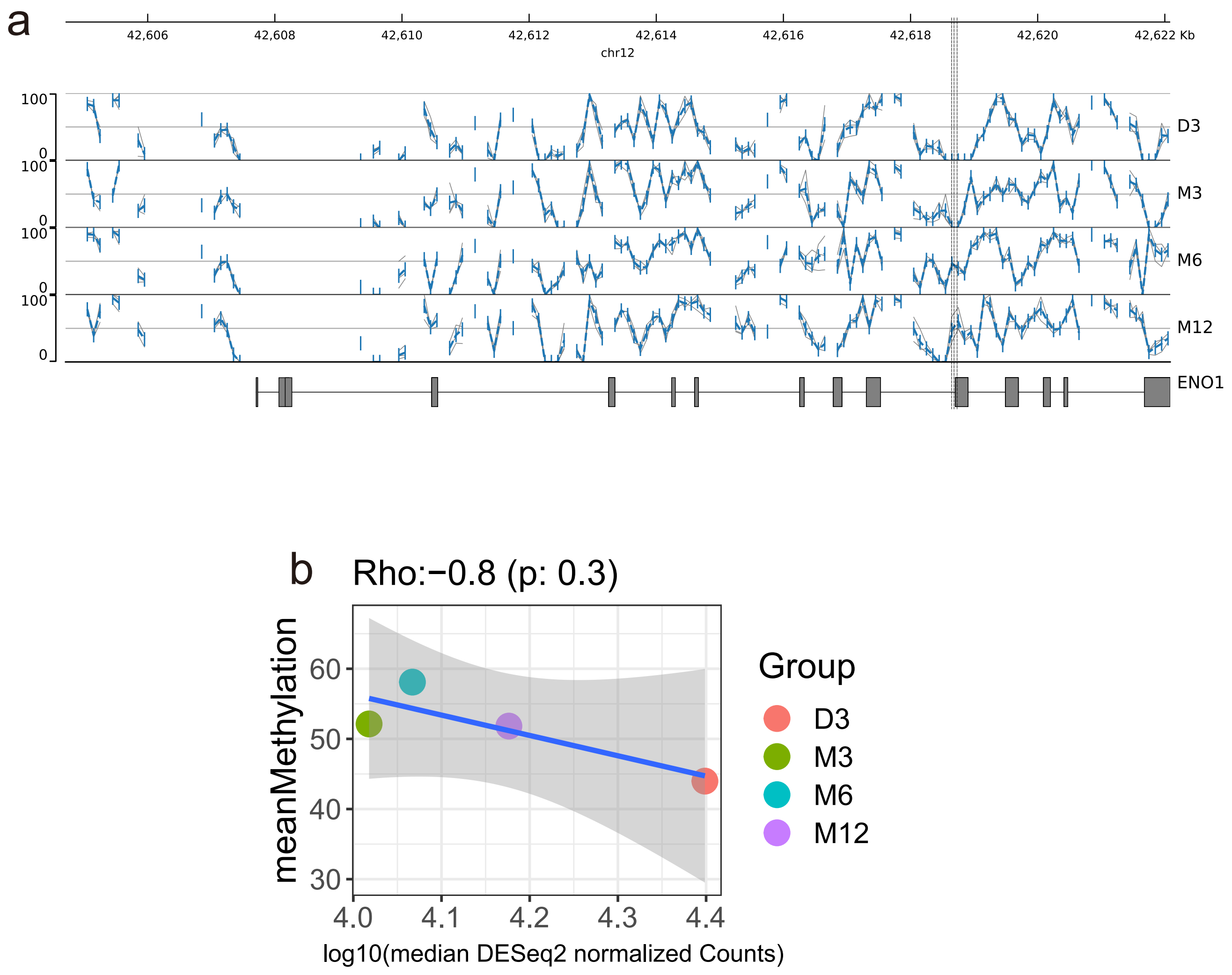


Supplementary Figure 6. **Methylation of *ENO1* and its correlation with transcription levels and methylation type**. (a) The methylation level on whole sequence was displayed and the dashed line indicated where the DMR is. GCF_000298735.2 was used for genome annotation. (b) The dot plot displayed the relationship between mean methylation on the *ENO1*'s body region and median expression of *ENO1*. Shading area indicated the 95% confidence intervalThe spearman correlation (rho) was -0.8, while the *p*-value is 0.3. DMR: differentially methylated regions.

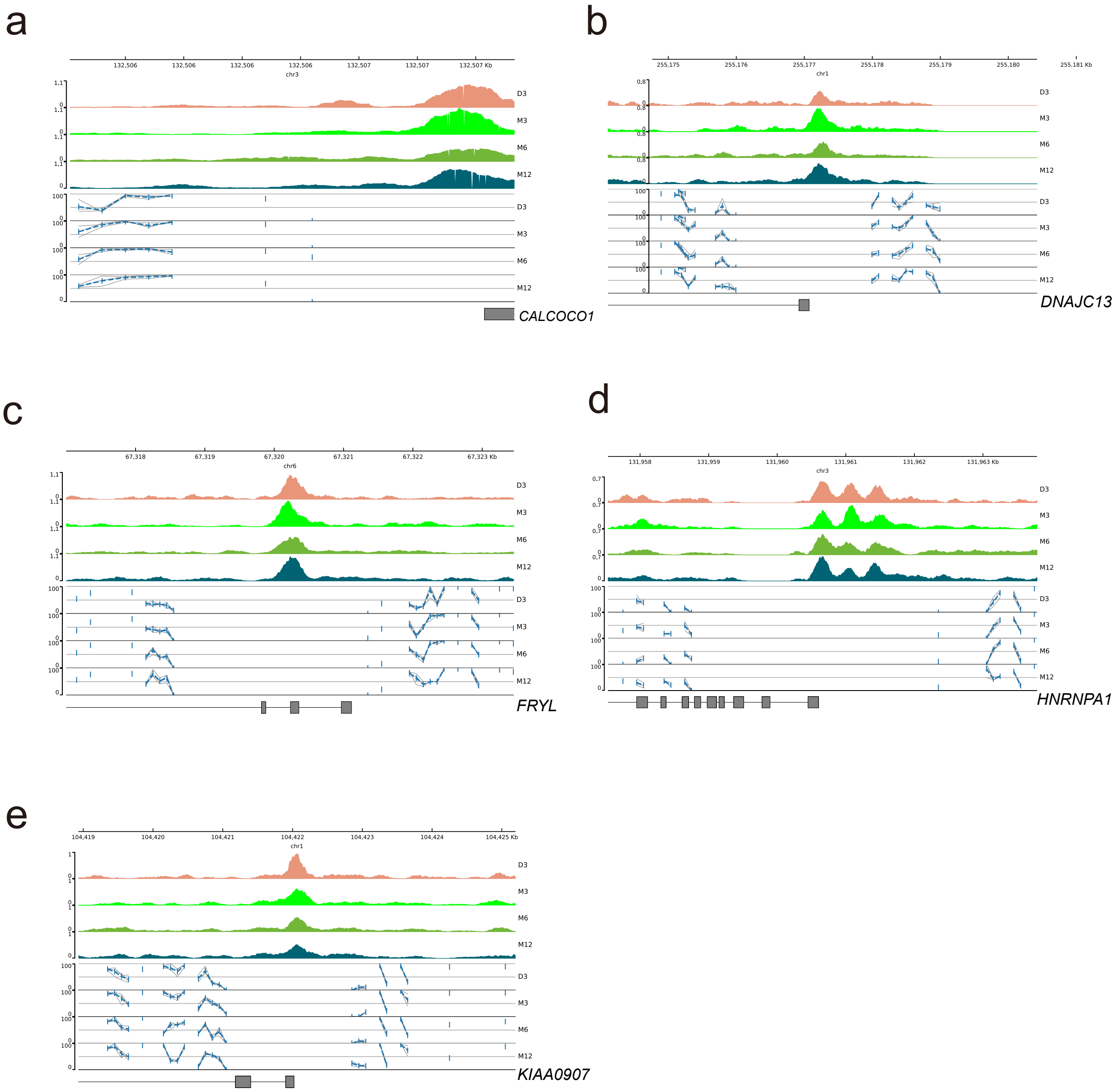


Supplementary Figure 7. **Candidate genes subjected to the interaction effect of accessible chromosome and methylation regions**. Genome browser of chromo-some accessibility and DNA methylation on promoter for *CALCOCO1* (a), *DNAJC13* (b), *FRYL* (c), *HNRNPA1* (d) and *KIAA0907* (e). GCF_000298735.2 was used for genome annotation.
