# Supplementary material for: Genome-wide epigenetic dynamics during postnatal skeletal muscle growth in Hu sheep
Source: Commun Biol. 2023 Oct 23;6:1077. doi: 10.1038/s42003-023-05439-0 (PMC10593826; doi:10.1038/s42003-023-05439-0)
Supplement: Supplementary file 2 — Description of additional supplementary files [file 42003_2023_5439_MOESM2_ESM.docx]

Description of Additional Supplementary Files

**File name:** Supplementary Data 1

**Description:** The source data behind the graphs in the paper

**File name:** Supplementary Data 2

**Description:** DEG of the pairwise comparisons in D3, M3, M6, M12

**File name:** Supplementary Data 3

**Description:** Summary statistics for ATAC-seq samples and IDR peak annotations.

**File name:** Supplementary Data 4

**Description:** Average methylation of gene across four stages.

**File name:** Supplementary Data 5

**Description:** Metadata of public WGS and ChIP-seq data
