# Supplementary material for: Genome-wide epigenetic dynamics during postnatal skeletal muscle growth in Hu sheep
Source: Commun Biol. 2023 Oct 23;6:1077. doi: 10.1038/s42003-023-05439-0 (PMC10593826; doi:10.1038/s42003-023-05439-0)
Supplement: Supplementary file 4 — Reporting Summary [file 42003_2023_5439_MOESM4_ESM.pdf]

nature portfolio

Corresponding author(s): Hongbing Han

Last updated by author(s): Sep 24, 2023

Reporting Summary

Nature Portfolio wishes to improve the reproducibility of the work that we publish. This form provides structure for consistency and transparency in reporting. For further information on Nature Portfolio policies, see our Editorial Policies and the Editorial Policy Checklist.

Statistics

For all statistical analyses, confirm that the following items are present in the figure legend, table legend, main text, or Methods section.

n/a	Confirmed	
☐	☒	The exact sample size (*n*) for each experimental group/condition, given as a discrete number and unit of measurement
☐	☒	A statement on whether measurements were taken from distinct samples or whether the same sample was measured repeatedly
☐	☒	The statistical test(s) used AND whether they are one- or two-sided<br>*Only common tests should be described solely by name; describe more complex techniques in the Methods section.*
☒	☐	A description of all covariates tested
☐	☒	A description of any assumptions or corrections, such as tests of normality and adjustment for multiple comparisons
☐	☒	A full description of the statistical parameters including central tendency (e.g. means) or other basic estimates (e.g. regression coefficient) AND variation (e.g. standard deviation) or associated estimates of uncertainty (e.g. confidence intervals)
☐	☒	For null hypothesis testing, the test statistic (e.g. *F*, *t*, *r*) with confidence intervals, effect sizes, degrees of freedom and *P* value noted<br>*Give P values as exact values whenever suitable.*
☒	☐	For Bayesian analysis, information on the choice of priors and Markov chain Monte Carlo settings
☒	☐	For hierarchical and complex designs, identification of the appropriate level for tests and full reporting of outcomes
☐	☒	Estimates of effect sizes (e.g. Cohen's *d*, Pearson's *r*), indicating how they were calculated

Our web collection on statistics for biologists contains articles on many of the points above.

Software and code

Policy information about availability of computer code

Data collection	For phenotype, original images were captured at 20X objective magnification using ECHO microscope (American).<br>For RNA-seq,libraries of six Hu sheep were prepared, and were sequenced on the Illumina platform for high-throughput sequencing with read PE150 (Frasergen Bioinformatics, Wuhan, China).<br>For ATAC-seq,libraries of three Hu sheep were prepared and the next generation high throughput sequencing was performed (Frasergen Bioinformatics, Wuhan, China).<br>Following fetual ChIP-seq samples were download: H3K4me3 samples (SRR5070525-SRR5070530), H3K27ac samples (SRR5070519-SRR5070524) and nucleosomal DNA (SRR5070531).<br>For WGBS,qualified library arrangement of three Hu sheep were sequenced (Frasergen Bioinformatics, Wuhan, China)□ 6 WGS samples (SRR10821772, SRR11657579-SRR11657583) were downloaded from NCBI SRA database .
Data analysis	For phenotype, we used Python and OpenCV to compute the CSA of muscle fibers. The calculation of slow-twitch muscle proportion using Image-Pro Plus 6.0 software. All statistics were analyzed using spss.<br>Trim_galora are used for filtering, including removal of adapters and low-quality reads. The filtered data is called clean data. Clean data was aligned with sheep reference genome by different aligners.<br>For RNA-seq data, a total of 242G sequencing data was obtained, with an average Q30 of 92%. Hisat2 2.2.1 was used for alignment. Then BAM files sorted by samtools 1.9 and quantified by featureCounts of subread 2.0.1. The DEG are obtained by DESeq2 1.26.0, with criteria as |Log2FoldChange| > 0 and qvalue < 0.05.<br>For ATAC-seq and ChIP-seq, clean data was aligned by Bowtie2 2.35.1, Samamba 0.6.6 is used to remove PCR duplicates. Use MACS2 2.1.0 to obtain the signal peak of the open area, with effective genome size set as 2.20E+06. For ATAC-seq data, parameter was set as --keep-dup=all --cutoff-analysis -g 2.20E+06 -B --SPMR --nomodel --shift -75 --extsize 150; For ChIP-seq , Parameter was set as --keep-dup all --nomodel --extsize 100 -g 2.20E+06 in MACS2. To get the consistent accessible peaks, use IDR(Irreproducible Discovery Rate) method within the repeated

samples, and use BEDTools intersect -wo to get the intersection.
For WGBS data, Bismark 0.23.0 software which calls Bowtie2 aligns the clean data of WGBS data to the reference genome, use deduplicate_bismark to remove duplicate reads, and use bismark_methylation_ extractor function to quantify methylation. Bulk DNA sample was used, so the methylation level of the cytosine base ranges from 0 to 100.
For WGS data, the following SNP calling process: 1) fastqc for data quality control; 2) BWA MEME 2.0 (doi.org/10.48550/arXiv.1303.3997) for alignment; 3) Samtools for sorting, indexing, and using Samamba to remove PCR duplications; 4) using HaplotypeCaller, CombineGVCFs, SelectVariants, VariantFiltration built in GATK4 4.1.8 to screen SNP mutation types and get high quality mutations (QUAL > 30). In order to better adapt to the data of this study, SNPs was also called base on WGBS data by Biscuit 0.3.16 (github.com/huishenlab/biscuit). Intersected loci were used for dowmstream analysis.

For manuscripts utilizing custom algorithms or software that are central to the research but not yet described in published literature, software must be made available to editors and reviewers. We strongly encourage code deposition in a community repository (e.g. GitHub). See the Nature Portfolio guidelines for submitting code & software for further information.

Data

Policy information about availability of data

All manuscripts must include a data availability statement. This statement should provide the following information, where applicable:

- Accession codes, unique identifiers, or web links for publicly available datasets
- A description of any restrictions on data availability
- For clinical datasets or third party data, please ensure that the statement adheres to our policy

The all data in this study are available within the supplementary information files, and multi-omics data reported in this paper will be shared by the lead contact upon request.

Human research participants

Policy information about studies involving human research participants and Sex and Gender in Research.

Reporting on sex and gender	*Use the terms sex (biological attribute) and gender (shaped by social and cultural circumstances) carefully in order to avoid confusing both terms. Indicate if findings apply to only one sex or gender; describe whether sex and gender were considered in study design whether sex and/or gender was determined based on self-reporting or assigned and methods used. Provide in the source data disaggregated sex and gender data where this information has been collected, and consent has been obtained for sharing of individual-level data; provide overall numbers in this Reporting Summary. Please state if this information has not been collected. Report sex- and gender-based analyses where performed, justify reasons for lack of sex- and gender-based analysis.*
Population characteristics	*Describe the covariate-relevant population characteristics of the human research participants (e.g. age, genotypic information, past and current diagnosis and treatment categories). If you filled out the behavioural & social sciences study design questions and have nothing to add here, write "See above."*
Recruitment	*Describe how participants were recruited. Outline any potential self-selection bias or other biases that may be present and how these are likely to impact results.*
Ethics oversight	*Identify the organization(s) that approved the study protocol.*

Note that full information on the approval of the study protocol must also be provided in the manuscript.

Field-specific reporting

Please select the one below that is the best fit for your research. If you are not sure, read the appropriate sections before making your selection.

☒ Life sciences ☐ Behavioural & social sciences ☐ Ecological, evolutionary & environmental sciences

For a reference copy of the document with all sections, see nature.com/documents/nr-reporting-summary-flat.pdf

Life sciences study design

All studies must disclose on these points even when the disclosure is negative.

Sample size	Sample sizes were chosen based on preliminary data demonstrating statistically significant differences for each specific assay. For phenotype and RNA-seq, the quadriceps femoris tissues of the six Hu sheep were obtained by local operation at the four developmental stages (D3, M3, M6, and M12), For ATAC-seq and WGBS data, the quadriceps femoris tissues of the three Hu sheep were obtained by local operation at the four developmental stages (D3, M3, M6, and M12),
Data exclusions	Full details of data exclusions for each analysis cn be found in the Methods as well.
Replication	All experiments were performed with at least three biological replicates on more than one occasion to ensure reproducibility across

Replication	experiments.
Randomization	Randomization and covariates were not relevant to our study design as we investigated single factors within each study.
Blinding	Blinding was not relevant as all processing methods were done through available software with consistent parameters utilized across all treatment groups.

Reporting for specific materials, systems and methods

We require information from authors about some types of materials, experimental systems and methods used in many studies. Here, indicate whether each material, system or method listed is relevant to your study. If you are not sure if a list item applies to your research, read the appropriate section before selecting a response.

Materials & experimental systems

n/a	Involved in the study
☒	☐ Antibodies
☒	☐ Eukaryotic cell lines
☒	☐ Palaeontology and archaeology
☐	☒ Animals and other organisms
☒	☐ Clinical data
☒	☐ Dual use research of concern

Methods

n/a	Involved in the study
☒	☐ ChIP-seq
☒	☐ Flow cytometry
☒	☐ MRI-based neuroimaging

Animals and other research organisms

Policy information about studies involving animals; ARRIVE guidelines recommended for reporting animal research, and Sex and Gender in Research

Laboratory animals	The six Hu sheep (three males and three females) were normally developed, vital, and without visible defects. At the four developmental stages (D3, M3, M6, and M12), the quadriceps femoris tissues of these six Hu sheep were obtained by local operation. In order to reduce the damage and avoid the impact of surgery, the left leg was operated at D3 and M6, while the right leg was handled at M3 and M12.
Wild animals	No wild animals were used.
Reporting on sex	The six Hu sheep (three males and three females) were selected in the experiment.
Field-collected samples	No Field-collected samples were used.
Ethics oversight	All experimental animal protocols were approved by the Animal Care and Use Committee at China Agricultural University (Aw03602202-1-1).

Note that full information on the approval of the study protocol must also be provided in the manuscript.
